# Cell surface interactome analysis identifies TSPAN4 as a negative regulator of PD‐L1 in melanoma

**DOI:** 10.1002/1878-0261.70182

**Published:** 2026-01-12

**Authors:** Guus A. Franken, Andrea Abel Gutierrez, Imke van Rossum, Cornelia G. Spruijt, Michiel Vermeulen, Guido van Mierlo, Blanca Scheijen, Annemiek B. van Spriel

**Affiliations:** ^1^ Department of Medical BioSciences, Radboud Institute for Medical Innovation Radboud University Medical Center Nijmegen The Netherlands; ^2^ Department of Molecular Biology, Faculty of Science Radboud University Nijmegen The Netherlands; ^3^ Oncode Institute Utrecht The Netherlands; ^4^ Division of Molecular Genetics The Netherlands Cancer Institute Amsterdam The Netherlands; ^5^ Department of Pathology Radboud University Medical Center Nijmegen The Netherlands; ^6^ BioSR Consultancy Eindhoven The Netherlands

**Keywords:** cell surface proximity biotinylation, melanoma, PD‐L1, TSPAN4

## Abstract

PD‐L1 is a key immune checkpoint ligand that suppresses antitumor immunity by engaging PD‐1 on T cells. While therapeutic blockade of PD‐L1/PD‐1 interactions has shown clinical benefit, many patients fail to respond, indicating modulation by other factors. Here, we identified a novel regulatory axis in which the membrane‐organizing protein tetraspanin‐4 (TSPAN4) modulates PD‐L1 in melanoma cells. Using cell surface proximity biotinylation coupled with mass spectrometry, we discovered that TSPAN4 physically associates with PD‐L1, with both proteins colocalizing on migrasomes and retraction fibers. Mechanistically, we show that TSPAN4 negatively regulates PD‐L1 protein levels by enhancing its degradation and restricting its lateral mobility at the plasma membrane. Loss of TSPAN4 stabilized PD‐L1, promoted its interaction with CMTM6, and increased PD‐L1 surface availability for PD‐1 binding. Functionally, TSPAN4 knockdown in melanoma cells led to more efficient immune checkpoint blockade through PD‐1 on T cells. This study identifies TSPAN4 as a negative regulator of PD‐L1 at the cell surface of melanoma cells suggesting that targeting TSPAN4 may offer a new therapeutic strategy to enhance immune checkpoint blockade in melanoma and other cancers.

## Graphical abstract

Using cell surface proximity biotinylation, we identified tetraspanin TSPAN4 within the PD‐L1 interactome of melanoma cells. TSPAN4 negatively regulates PD‐L1 expression and lateral mobility by limiting its interaction with CMTM6 and promoting PD‐L1 degradation. TSPAN4 impacts PD‐1 binding and T‐cell inhibition revealing TSPAN4 as a novel regulator of PD‐L1 function and a promising therapeutic target.

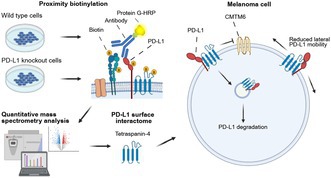

**Abbreviations**
APC: antigen‐presenting cell
Cas9: CRISPR‐associated protein 9
CD: cluster of differentiation (e.g., CD80, CD86)
CMTM6: CKLF‐like MARVEL transmembrane domain‐containing 6
ELISA: enzyme‐linked immunosorbent assay
FACS: fluorescence‐activated cell sorting
IF: immunofluorescence
IFNγ: interferon gamma
IL‐2: interleukin 2
KD: knockdown
KO: knockout
MFI: median fluorescence intensity
MHC: major histocompatibility complex
mRNA: messenger ribonucleic acid
NT: nontargeting (control)
PBS: phosphate‐buffered saline
PD‐L1: programmed death‐ligand 1
TCR: T‐cell receptor
TSPAN4: tetraspanin 4
WT: wild‐type

## 1 Introduction

Tumor cells can be recognized by the immune system through the expression of tumor‐specific neo‐antigens [1]. Co‐inhibitory checkpoint ligands on tumor cells inhibit these immune responses, exemplified by programmed death‐ligand 1 (PD‐L1) that binds programmed cell death protein 1 (PD‐1) expressed on immune cells, including T lymphocytes [2, 3]. Ligation of PD‐L1 to PD‐1 results in SHP‐2 mediated inhibition of T‐cell receptor (TCR) and CD28 signaling, leading to dampened T‐cell activation and tumor immune evasion [4, 5, 6]. Blockade of PD‐L1 using monoclonal antibodies such as durvalumab relieves T‐cell suppression and has been shown to enhance clinical outcomes across multiple cancer types, including melanoma. However, in many cases treatment responses are limited, indicating that more insight into the molecular mechanisms modulating immune checkpoint therapy efficacy is required [7, 8, 9].

The biophysical properties of PD‐L1 and its interacting proteins at the plasma membrane are incompletely understood. PD‐L1 binds to the costimulatory ligand CD80 in *cis*, which alleviates T‐cell inhibition by preventing PD‐L1 interaction with PD‐1 in *trans* [10]. Furthermore, the intracellular domain of PD‐L1 seems to be a major factor involved in potentiating PD‐L1 dimerization [11] and stability through electrostatic membrane association [12], while preventing interferon‐mediated toxicity through reverse signaling [13]. Notably, PD‐L1 may also dimerize with its paralogous protein PD‐L2 [14]. PD‐L1 is predominantly present on the cell surface and dynamically cycles between endosomal and plasma membrane localization as part of its homeostatic regulation [15, 16, 17, 18]. Recent studies have identified the PD‐L1 interacting protein CKLF‐like MARVEL transmembrane domain‐containing member 6 (CMTM6), which promotes PD‐L1 endosomal recycling to the plasma membrane and limits lysosomal degradation [15, 19, 20]. Despite significant research into the intracellular trafficking of PD‐L1, its lateral mobility within the plasma membrane remains poorly understood.

The lateral mobility of membrane proteins varies [21, 22] and is regulated by different factors, including intrinsic and extrinsic membrane‐organizing factors [23]. Restriction of protein mobility by the cytoskeleton (as proposed in ‘the picket‐fence model’) is an example of an extrinsic membrane‐organizing factor [24]. Furthermore, proteins can be organized on the plasma membrane by intrinsic factors like lipid rafts and tetraspanins. Tetraspanins are a family of 33 four‐transmembrane proteins that exert their function by influencing the expression, mobility, and/or activity of interaction partners [25, 26, 27].

In this study, we applied cell surface proximity biotinylation combined with mass spectrometry to detect new PD‐L1 interacting proteins to better understand the regulation of PD‐L1 at the plasma membrane of tumor cells. Cell surface proximity biotinylation is an enzymatic labeling technique in which an antibody is coupled to an enzyme that produces short‐lived reactive biotin radicals at the immediate vicinity of the antibody binding site on living cells. Biotinylated proteins can be captured by streptavidin and identified by mass spectrometry [28, 29]. This technology allows for unraveling the cell surface interactome of PD‐L1 under normal physiological conditions in living cells. Our results identified tetraspanin superfamily member TSPAN4 as a novel interacting protein of PD‐L1 in melanoma cells, which negatively regulates PD‐L1 cell surface expression levels and lateral mobility.

## 2 Materials and methods

### 2.1 Cell culture

BLM (obtained from AIMM Therapeutics, RRID:CVCL_7035) and Mel624 (obtained from ATCC, RRID:CVCL_8054) cells were maintained in Dulbecco's modified Eagle medium (DMEM) (Thermo Fisher Scientific, Waltham, MA, USA, #31966‐021) supplemented with 10% FBS (Cytiva, Marlborough, MA, USA, #SV30160.03) and 1% antibiotic‐antimycotic (Gibco, Thermo Fisher Scientific, #15240062) (DMEM CM). For siRNA transfections, DMEM was used without phenol red (Thermo Fisher Scientific, #31053‐028) and antibiotic‐antimycotic. Jurkat T cells were maintained in Roswell Park Memorial Institute (RPMI) 1640 Medium (Thermo Fisher Scientific, #42401‐018) supplemented with 1% stable glutamine (Capricorn, Ebsdorfergrund, Germany, #STA‐B), 10% FBS, and 1% antibiotic‐antimycotic (Gibco, Thermo Fisher Scientific, #15240062) (RPMI CM). Cells were cultured in a humidified incubator at 37 °C and 5% CO_2_ and were authenticated in the past 3 years by DNA profiling (STR). Cells were regularly checked for mycoplasma contamination, and all experiments were conducted in mycoplasma‐free cells.

### 2.2 Flow cytometry

BLM and Mel624 cells were detached with 1.5 mm EDTA in PBS for 5 min at 37 °C, harvested and plated in v‐bottom FACS plates, and stained with primary antibodies or recombinant human PD‐1‐Fc chimera protein (BioLegend, San Diego, CA, USA, #785106 for Mel624, Thermo Fisher Scientific, #A42530 for BLM) and secondary antibodies for 30 min at 4 °C in PBA (PBS + 1%BSA + 0.05% sodium azide). Staining with LIVE/DEAD™ Fixable Aqua Dead Cell Stain Kit (Thermo Fisher Scientific, #L34957) for 15 min at 4 °C was used for discerning live from dead cells. For intracellular staining, cells were fixed with 4% paraformaldehyde (PFA) in PBS for 20 min at RT and permeabilized and quenched with 0.1% triton X‐100 (Sigma‐Aldrich, Burlington, MA, USA, # X100) and 50 mm glycine (Thermo Fisher Scientific, # ICN808831) in PBS for 10 min at RT.

For the flow cytometric‐based degradation assay, cells were stained with primary conjugated antibodies for 30 min at 4 °C in DMEM CM, washed 3× with ice‐cold PBS, resuspended in warm DMEM CM and divided in a low‐attachment 96‐well plate, and incubated at 37 °C and 5% CO_2_. At indicated timepoints, cells were collected and placed in cold DMEM CM at 4 °C. After the final incubation step, cells were stained with LIVE/DEAD™ Fixable Aqua Dead Cell Stain Kit in PBS for 15 min at 4 °C for discerning live from dead cells and washed in PBA.

Primary antibodies used: mouse anti‐PD‐L1‐APC (BioLegend, #329708, 1 : 40), mouse anti HLA‐ABC‐PE (BioLegend, #311406, 1 : 100), mouse anti‐PD‐L1 (Thermo Fisher Scientific, #14‐5983‐82, 1 : 50), mouse anti‐PD‐L2 (Thermo Fisher Scientific, #14‐5888‐82, 1 : 50), mouse anti‐PD‐1‐PE (BD Biosciences, San Jose, CA, USA, #557946, 1:20), mouse anti CD8‐PE/Cy7 (BioLegend, #344750, 1 : 20), mouse anti HLA‐ABC (Thermo Fisher Scientific, #14‐9983‐82, 1 : 50), mouse IgG1 isotype (BioLegend, #400102, 1 : 50), mouse IgG2b‐APC isotype (Thermo Fisher Scientific, #17‐4732‐42, 1 : 40), mouse IgG2a‐PE isotype (BD Biosciences, #349053, 1 : 20), mouse IgG1‐PE isotype (BioLegend, #400112, 1 : 20), mouse IgG1‐PE/cy7 isotype (BD Biosciences, #557872, 1 : 20). Secondary antibodies used: goat anti mouse IgG1‐Alexa647 (Thermo Fisher Scientific, #A21240, 1 : 400), goat anti mouse IgG2a‐Alexa488 (Thermo Fisher Scientific, # A21131, 1 : 400). Analysis was done with FACSVerse or FACSLyric flow cytometers (BD Biosciences), and data were analyzed using flowjo x Software (FlowJo v10.0.7r2, FlowJo LLC, Ashland, OR, USA).

### 2.3 Plasmids

The plasmids PD‐L1‐FLAG, TSPAN4‐GFP, PD‐1‐FLAG, and CD81‐GFP were obtained from Genscript (Piscataway, NJ, USA), and the PX459 Cas9 vector was obtained from Addgene (Watertown, MA, USA). The C‐terminal ALFA‐tagged [30] TSPAN4 and CD81 constructs were generated from TSPAN4‐GFP and CD81‐GFP by the Q5 Site‐Directed Mutagenesis Kit (#E0554S, New England Biolabs, Ipswich, MA, USA) according to the manufacturer's protocols and using custom‐designed primers (Sigma‐Aldrich). All constructs were verified by Sanger sequencing.

### 2.4 DNA transfection

For co‐IP experiments, 2 × 10^6^ BLM wild‐type (WT) cells were seeded in T75 flasks 3 days before transfection. One day before co‐IP, cells were transfected by adding 650 μL Opti‐mem (Thermo Fisher Scientific, #11058‐021) containing 6.5 μg DNA and 31.25 μL PEI (Polysciences, Warrington, PA, USA, #24765) that was pre‐incubated for 15 min at RT. For IF experiments, 4 × 10^4^ BLM WT cells were seeded on 12 mm #1.5 thickness coverslips 1 day before transfection. Cells were transfected by adding 50 μL Opti‐mem containing 0.5 μg DNA and 2.4 μL PEI that was pre‐incubated for 15 min at RT. For investigating the effect of TSPAN4‐ALFA and CD81‐ALFA on PD‐L1 expression, 3 × 10^5^ BLM and Mel624 cells were seeded in 6 well plates 1 day before transfection. On the day of transfection, cells were incubated with 50 ng·mL^−1^ IFNγ (Gibco, Thermo Fisher Scientific, #P01579) and transfection was performed by adding 250 μL Opti‐mem containing 2.5 μg DNA, 5 μL P3000, and 6 μL Lipofectamine 3000 that was pre‐incubated for 15 min at RT. All transfections were performed on subconfluent densities, and for co‐IP, IF, and FACS experiments, cells were processed 1 day after transfection.

### 2.5 BLM PD‐L1 and PD‐L2 KO generation

BLM WT cells were transfected with the PX459 Cas9 vector (Addgene) containing a guide RNA sequence targeting *PD‐L1* (oligo sense: CACCGCATAGTAGCTACAGACAGA, oligo anti‐sense: AAACTCTGTCTGTAGCTACTATGC) or the *PD‐L2* gene (oligo sense: CACCGCCAGGCTCAACATTAGCAGG, oligo anti‐sense: AAACCCTGCTAATGTTGAGCCTGGC), which were obtained from Sigma‐Aldrich and designed using CRISPOR.org. One day after transfection, untransfected cells were excluded by 1 μg·mL^−1^ puromycin treatment for 2 days, after which cells were limitedly diluted and grown from monoclonal colonies (for PD‐L1 KO cells) or sorted with FACSMelody (BD Biosciences) after fluorescently staining cell surface PD‐L2 and gating on PD‐L2‐negative cells (for PD‐L2 KO cells). Knockout was validated by flow cytometry staining and genomic DNA isolation (Qiagen, Hilden, Germany) followed by PCR of the gene of interest using primers (Sigma‐Aldrich) and Sanger sequencing.

### 2.6 Proximity biotinylation and pull down with streptavidin beads

BLM WT and PD‐L1 KO cells were seeded at 3 × 10^6^ cells in T175 flasks 3 days before experiments, cultured with 50 ng·mL^−1^ IFNγ 1 day before being detached with 1.5 mm EDTA in PBS for 5 min at 37 °C. 15 × 10^6^ cells were resuspended in PBS + 0.5% BSA and stained for 30 min on ice with 5 μg·mL^−1^ anti‐PD‐L1 antibody (Thermo Fisher Scientific, #14‐5983‐82) or mouse IgG1 isotype antibody (BioLegend, #400102) that was pre‐incubated for 1 h on ice with 7 μg·mL^−1^ protein G‐horseradish peroxidase (HRP) (Merck Sigma‐Aldrich, #18‐161). Afterwards, cells were washed 3× with ice‐cold PBS and resuspended in biotin phenol solution (500 μm in PBS) (Sigma‐Aldrich, #SML2135‐50MG) for 1 min at RT and then an equal volume of H_2_O_2_ (2 mm) (Supelco, Bellefonte, PA, USA, #1072090250) in PBS was added, and cells were incubated for 4 min at RT. Then, ice‐cold quenching buffer (QB) (5 mm trolox (Sigma‐Aldrich, # 238813‐5G), 10 mm sodium ascorbate (Sigma‐Aldrich, #A7631), 10 mm sodium azide (Sigma‐Aldrich, # 8.22335) in PBS) was added, cells were put on ice, and cells were washed 3× with QB. Subsequently WT and PD‐L1 KO cells were lysed in lysis buffer (1% Brij97 (Sigma‐Aldrich, # P6136), 10 mm Tris/HCL (pH 7.5), 150 mm NaCl, 2 mm MgCl_2_, 2 mm CaCl_2_, 10% QB in Milli‐Q, supplemented with protease inhibitor (Roche, Basel, Switzerland, # 05892970001) and phosphatase inhibitor (Roche, # 4906845001)) with constant agitation at 4 °C for 45 min. For the a‐IgG/a‐PD‐L1 proximity biotinylation, cell lysis was performed overnight in 50 mm Tris/HCL (pH 7.5), 150 mm NaCl, 0.5% sodium deoxycholate (Sigma‐Aldrich, #D6750‐10G), 0.1% (wt/vol) SDS, and 1% (vol/vol) NP40 (Sigma‐Aldrich, #I8896‐50ML). Insoluble material was removed by 10 min 10 000 ***g*** centrifugation, after which biotinylated proteins were pulled down using streptavidin sepharose beads (Merck Sigma‐Aldrich, # GE17‐5113‐01) for 2 h at 4 °C under constant agitation. Beads were washed 5× with lysis buffer, proteins were eluted from beads using 2× reducing Laemmli sample buffer (Bio‐Rad, Hercules, CA, USA, # 1610747) at 95 °C for 10 min, and samples were stored at −20 °C.

### 2.7 Cell surface interactome analysis with mass spectrometry

For sample preparation, streptavidin beads from the proximity biotinylation assay were washed 4× with 1× PBS. After the last wash, all PBS was removed using a syringe and 50 μL elution buffer (2 m Urea, 10 mm DTT, 0.1 m Tris pH 8) was added to the beads, followed by incubation on a thermoshaker at 4 ***g*** for 20 min at RT. Iodoacetamide was added to a final concentration of 50 mm, and samples were incubated in a thermoshaker at 4 ***g*** in the dark for 10 min at RT. About 2.5 μL of trypsin (0.1 mg·mL^−1^ stock solution) was added to each sample, followed by incubation in a thermoshaker at 4 ***g*** for 2 h at RT. Samples were centrifuged, and the eluted fraction was collected. About 50 μL of additional elution buffer was added to the beads, and after incubation in a thermoshaker at 4 ***g*** for 5 min at RT, the eluates were combined. About 1 μL of fresh trypsin was added to each sample, followed by overnight incubation at RT. The next day peptides were acidified by adding 10 μL of 10% v/v TFA and purified on C18 Stagetips (3M Empore) [31].

The tryptic peptides from PD‐L1 KO and WT BLM cells were analyzed on a Orbitrap Exploris 480 (Thermo Fisher Scientific) on a 60‐min gradient in the data‐dependent acquisition mode. The Orbitrap resolution was set to 120 000, the scan range (m/z) at 350–1300, the normalized AGC Target to 300%, and the maximum injection time to 20 ms. Peptides from wild‐type BLM cells with proximity biotinylation performed using a‐IgG or a‐PD‐L1 antibodies were analyzed on an Orbitrap Astral (Thermo Fisher Scientific) on a 24‐min gradient in the data‐independent acquisition mode. The Orbitrap resolution was set to 240 000, the scan range (m/z) at 380–980, the normalized AGC Target to 500%, and the maximum injection time to 5 ms.

Raw spectra were analyzed using maxquant 1.5.1.0 [32] for Orbitrap Exploris data or dia‐nn [33] for spectra obtained using the Orbitrap Astral. perseus 1.5.0.15 was used to filter proteins flagged as contaminants [34]. Individual protein abundances were determined by label‐free quantification (LFQ). All experiments were performed in triplicate for each antibody, using an independent cell mixture for each replicate as input. The triplicates were grouped based on the condition (WT/KO or a‐IgG/a‐PD‐L1), and only proteins that had an LFQ value in each of the replicates in at least one group of triplicates were maintained for downstream analyses [35, 36]. Missing values were imputed using default parameters (width = 0.3, shift = 1.8) in perseus. Statistically different proteins were identified using a Student's *t*‐test (FDR < 0.05) between a‐PD‐L1 and control triplicates or PD‐L1 KO and WT BLM cells.

### 2.8 Immunofluorescence microscopy

Cells were harvested with 1.5 mm EDTA in PBS for 5 min at 37 °C, counted, and 4 × 10^4^ cells were seeded on sterile 12 mm #1.5 thickness coverslips in a 24‐well plate in DMEM CM. About 24 h after seeding, cells were treated with 50 ng·mL^−1^ IFNγ and transfected with DNA as described earlier. After another 24 h, coverslips were washed 2× with PBS and fixed with 4% PFA in PBS for 20 min at RT. Coverslips were blocked with blocking buffer (5% BSA + 0.3 m glycine + 2% human serum + 1% goat serum in PBS) for 30 min at RT and stained with primary and secondary antibodies in blocking buffer for 30 min at RT each, with PBS washes in between. For intracellular staining, cells were permeabilized with 0.1% triton X‐100 in PBS for 10 min at RT. Finally, cells were stained with 0.3 μg·mL^−1^ 4′‐6‐diamidino‐2‐phenylindole (DAPI) for 2 min at RT, washed with PBS and Milli‐Q, and embedded in Fluoromount G (Thermo Fisher Scientific, # 00‐4958‐02). Imaging was performed using a Zeiss LSM900 confocal microscope equipped with an Airyscan Detector and a 63× Plan‐Apochromat oil immersion 1.4NA objective with Zeiss zen software. Data analysis was conducted using fiji image analysis software. Pearson correlation analysis was performed using BIOP JACoP plugin. Antibodies used: rabbit anti‐FLAG (Merck, #F7425, 1 : 1000), mouse anti‐PD‐L1 (Thermo Fisher Scientific, #14‐5983‐82, 1 : 50), mouse anti‐CD55 (Thermo Fisher Scientific, # MA1‐91161, 1 : 100), anti‐ALFA‐AZDye568 (NanoTag Technologies, Göttingen, Germany, #N1502, 1 : 100). Secondary antibodies used: goat anti‐mouse IgG1‐Alexa647 (Thermo Fisher Scientific, #A21240, 1 : 400), goat anti‐rabbit IgG‐Alexa647 (Thermo Fisher Scientific, #A21245, 1 : 400).

### 2.9 Co‐immunoprecipitation

Cells were seeded in T75 or T175 flasks 2–3 days before co‐immunoprecipitation (co‐IP). Two days before IP of endogenous PD‐L1, cells were treated with 50 ng·mL^−1^ IFNγ. Cells were transfected with TSPAN4‐ALFA and PD‐L1‐FLAG 1 day before co‐IP experiments. Protein G sepharose beads (Cytiva, #17061801) were blocked with 3% BSA or incubated with 10 μg·mL^−1^ mouse IgG1 isotype antibody in PBS overnight. On the day of co‐IP, subconfluent cells were detached by 1.5 mm EDTA in PBS for 5 min at 37 °C, harvested with PBS, and centrifuged at 413 ***g*** for 5 min before being lysed in ice‐cold lysis buffer (1% Brij97, 10 mm Tris/HCL (pH 7.5), 150 mm NaCl, 2 mm MgCl_2_, 2 mm CaCl_2_ in MQ, supplemented with protease inhibitor and phosphatase inhibitor) for 45 min with constant agitation at 4 °C. Insoluble material was removed by 6 min 3800 ***g*** centrifugation. Protein G sepharose beads were washed with lysis buffer, and preclear was performed with BSA‐blocked and isotype‐coated protein G sepharose beads for 1 h at 4 °C with constant agitation. Thereafter, lysate was incubated with 3 μg antibody (rabbit anti‐FLAG (Merck, #F7425)) for 1 h at 4 °C with constant agitation, and subsequently with protein G sepharose beads for 2 h at 4 °C with constant agitation. For ALFA IP, after washing ALFA selector ST beads (NanoTag technologies, # N1511), beads were directly added to lysate without preclear for 2 h at 4 °C. Afterwards, beads were washed 5× with ice‐cold wash buffer (0.1% Brij97, 10 mm Tris/HCl (pH 7.5), 150 mm NaCl, 2 mm MgCl_2_, 2 mm CaCl_2_ in MQ, supplemented with protease inhibitor and phosphatase inhibitor). Proteins were eluted from beads using 2× reducing Laemmli sample buffer at 95 °C for 10 min and stored at −20 °C.

### 2.10 Western blotting

For western blot, protein samples were separated on a 12% SDS polyacrylamide gel and transferred to a PVDF membrane (GE Healthcare, Chicago, IL, USA). The blot was blocked with 5% ELK in TBS‐T or 5% BSA in PBS for 1 h at RT and incubated with primary antibodies (rabbit anti‐PD‐L1 (Cell Signaling Technology, Danvers, MA, USA, #13684S, 1 : 1000), rabbit anti CMTM6 (Atlas Antibodies, Stockholm, Sweden, #HPA026980, 1 : 1000), anti‐ALFA‐Alexa647 nanobody (NanoTag Technologies, #N1502, 1 : 1000)) overnight. After incubation with IRDye^®^ 680LT Streptavidin (LICORbio, Lincoln, NE, USA, #926‐68031) or secondary antibody for 1 h at RT, the blot was scanned using Amersham Typhoon™ (Cytiva). Washes were performed with TBS + 0.05% Tween‐20. Blots were analyzed using Image Studio Light (LICORbio). Secondary antibody used: IRDye^®^ 800CW Goat anti‐Rabbit IgG (LICORbio, # 925‐32211).

### 2.11 siRNA transfection

Cells were washed with PBS (Fresenius Kabi, Bad Homburg, Germany), detached with 1.5 mm EDTA in PBS for 5 min at 37 °C, harvested in DMEM CM, and counted. SiRNA complexes were prepared in 6 well plate wells by incubating 60 pmol TSPAN4 smartpool siRNA (Dharmacon, Lafayette, CO, USA, #L‐010625‐02‐0020) or NT smartpool siRNA (Dharmacon, #D‐001810‐10‐20) with 7.5 μL Lipofectamine 3000 (Thermo Fisher Scientific, #L3000015) in 250 μL Opti‐mem (Thermo Fisher Scientific,  #31985070) for 5 min at RT, after which 2 × 10^5^ BLM or Mel624 cells resuspended at 0.8 × 10^5^ cells·mL^−1^ in DMEM CM minus phenol red and minus antibiotic‐antimycotic were seeded on top in 6 well plate wells. After 3 days, cells were collected and replated in regular DMEM CM for further experiments.

### 2.12 RNA isolation, reverse transcription, and qPCR

For each isolation, 0.1–1 × 10^6^ cells were washed with PBS and resuspended in 1 mL TRIzol™ Reagent (Invitrogen, #15596026). Subsequently, 200 μL RNAse‐free chloroform (Emsure, Burlington, MA, USA, #1024451000) was added, incubated for 3 min, and after 15 min centrifugation at 18 000 ***g*** at 4 °C, the aqueous phase was taken for further processing. RNA was pelleted by adding 500 μL RNAase‐free iso‐propanol (Supelco, #PX1830) and 1 μL glycogen, incubating for 10 min at RT, centrifugation at 18 000 ***g*** at 4 °C, and removing the supernatant. The pellet was washed with RNAse‐free 75% ethanol (Supelco,  #1009831011) and centrifugation at 6000 ***g*** at 4 °C, air‐dried, and resuspended in RNAse‐free Milli‐Q (Invitrogen, #10977015). RNA concentration was measured by nanodrop, and DNAse treatment was performed on 2 μg RNA in a 25 μL total volume consisting of 2.5 μL DNAse buffer (Invitrogen, #18068‐015), 2.5 μL DNAse‐I (Invitrogen, #18068‐015), and RNase‐free Milli‐Q for 15 min at RT. The reaction was stopped by adding 2.5 μL EDTA (Invitrogen, #18068‐015) and incubation for 10 min at 68 °C. Reverse transcription was initiated by adding 1 μL 100 μm hexamers (Sigma‐Aldrich, #11034731001) and 1.2 μL 10 mm dNTPs (Eurogentec, #NU‐0020‐50) to 9.8 μL DNAse‐treated RNA solution, heated for 5 min at 65 °C, and put on ice afterwards. Then, 4 μL First‐strand buffer 5× (Invitrogen, #28025‐021), 2 μL DTT (Invitrogen, #28025‐021), 1 μL RNasin (Promega, Madison, WI, USA, #N2515), and 1 μL Reverse Transcriptase (Invitrogen, #28025‐021) were added to each sample and incubated for 10 min at 25 °C, 50 min at 37 °C, 15 min at 70 °C, and thereafter stored at −20 °C.

For qPCR, the thermocycling conditions were as follows: predenaturation at 94 °C for 5 min; 40 cycles of denaturation at 94 °C for 30 s, annealing at 60 °C for 20 s, and extension at 72 °C for 20 s; and a final extension at 72 °C for 10 min. All RT‐qPCR reactions yielded products with a single dissociation peak. Results were analyzed using the 2 − ΔΔCq method. SYBR green (Applied Biosystems, Waltham, MA, USA, #A25742) was used according to the manufacturer's instructions. Primers used for *TSPAN4* qPCR were CGTCAAGTACCTCATGTTC (FW), ACGGGAAGGAAGAGGAC (RV), for *GAPDH* GAAGGTGAAGGTCGGAGTC (FW), GAAGATGGTGATGGGATTTC (RV).

### 2.13 Fluorescence recovery after photobleaching

The day before fluorescent recovery after photobleaching (FRAP) was performed, BLM TSPAN4 KD and NT cells were seeded at 4 × 10^5^ cells in Willco dishes in DMEM CM. On the day of FRAP, cells were washed with Leibovitz medium (Thermo Fisher Scientific,  #21083027) and incubated with 10 μg·mL^−1^ anti‐PD‐L1 Alexa‐488 antibody (Thermo Fisher Scientific, #53‐5983‐42, 1 : 100) in Leibovitz medium for 15 min at 37 °C, after which cells were washed 3× and incubated with Leibovitz medium. FRAP experiments were performed with a Leica TCS SP8 SMD microscope equipped with a 60× water 1.2 NA objective (Leica) and an argon‐ion laser set to bleach with 100% power at the 488 nm wavelength. The fluorescence intensity in the bleach zone as well as the whole cell and background was measured to correct for photobleaching and background signal. Immobile and mobile fractions, as well as the recovery curve and speed of recovery (T‐half), were calculated manually and confirmed using the easyfrap web tool [37].

### 2.14 Stable PD‐1‐overexpressing Jurkat T‐cell line generation

For experiments with Jurkat T cells stably expressing PD‐1, we used a previously generated Jurkat T‐cell line that stably expressed full‐length 296 TCRαβ specific for the gp100 peptide and CD8α after retroviral transduction as described in [38]. In order to obtain a Jurkat T‐cell population stably expressing PD‐1, 2 × 10^6^ Jurkat T cells were transfected with 2 μg PD‐1‐FLAG DNA using the Neon transfection system according to the manufacturer's instructions, and starting 2 days after transfection for a period of 3 weeks, cells were sorted with FACSMelody after fluorescently labeling cell surface PD‐1 (mouse anti‐PD‐1‐PE (BD Biosciences, #557946, 1 : 20)) and gated on PD‐1‐positive cells.

### 2.15 Jurkat coculture assays

BLM and Mel624 cells were seeded 2 days before the Jurkat coculture assay in T25 flasks with 50 ng·mL^−1^ IFNγ. At the day of the assay, cells were detached with 1.5 mm EDTA in PBS, counted, and resuspended in DMEM CM without FBS at 1 × 10^6^ cells·mL^−1^ containing 1 μg·mL^−1^ gp100 peptide (JPT Peptide Technologies, Berlin, Germany, #SP‐MHCI‐0084‐2), incubated for 1 h at 37 °C, washed 3× with RPMI CM, and resuspended in RPMI CM. 4 × 10^4^ cells were seeded per well in 96‐well plates and incubated with 4 μg·mL^−1^ durvalumab (Imfinzi, Medimmune/AstraZeneca, provided by RadboudUMC pharmacy) or human IgG1 isotype antibody (BioLegend, #403501) for 30 min at 37 °C. Jurkat T cells were collected, counted, and resuspended in fresh RPMI CM containing 4 μg·mL^−1^ CD28 antibody (InVivoMAb, Lebanon, NH, USA, # BE0248), and 2 × 10^5^ cells were added on top of the BLM and Mel624 cells and incubated at 37 °C. After 24 h, supernatant was collected and IL‐2 ELISA (Thermo Fisher Scientific, #88‐7025‐88) was performed.

### 2.16 Survival analysis

GEPIA 2 (http://gepia2.cancer‐pku.cn/) was used for comparing overall survival between *PD‐L1* and *TSPAN4* high and low expressing skin cutaneous melanoma patients based on mRNA levels from the TCGA Skin Cutaneous Melanoma (SKCM) cohort (*n* = 470) [39, 40]. The majority of cases involved patients with metastatic disease (364 out of 470 cases) at the time of diagnosis.

### 2.17 Statistical analysis

Statistical analysis was performed using graphpad prism 9 (GraphPad, San Diego, CA, USA). Normality was assessed using the Shapiro–Wilk test. subcellularvis [41] was used for determining the significance of subcellular localization of WT‐enriched proteins of mass spectrometry data.

## 3 Results

### 3.1 Identification of PD‐L1 interacting proteins in melanoma by proximity biotinylation followed by mass spectrometry

**Fig. 1:**
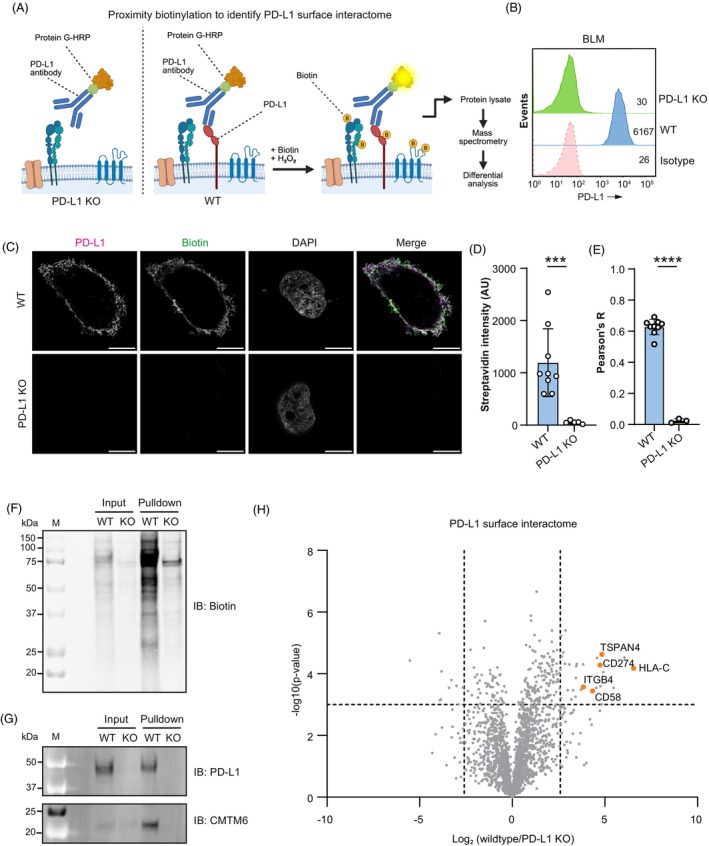
Identification of PD‐L1 interacting proteins in melanoma by proximity biotinylation followed by mass spectrometry. (A) Schematic overview of surface proximity biotinylation assay to identify PD‐L1 interacting proteins at the plasma membrane. PD‐L1 is stained on the cell surface with a PD‐L1 antibody coupled to protein G‐horseradish peroxidase (HRP), which forms biotin radicals upon addition of biotin and hydrogen peroxide. After proximity biotinylation cells are lysed and biotinylated proteins are pulled down with streptavidin beads and analyzed by mass spectrometry. Created in BioRender. Franken, G. (2025) https://BioRender.com/9fluxpk. (B) Flow cytometry PD‐L1 surface expression histograms of wild‐type (WT) and PD‐L1 KO BLM cells, representative for *n* = 3 biological replicates. (C) Immunofluorescence (IF) images of BLM and PD‐L1 KO cells stained for PD‐L1, biotin, and DAPI after PD‐L1 surface proximity biotinylation, representative for *n* = 3 biological replicates. Scale bar: 10 μm. (D, E) Quantification of biotin signal (D) and Pearson correlation coefficient between biotin and PD‐L1 (E) from IF staining after surface proximity biotinylation. Error bars indicate mean ± SD. Significance was determined by Mann–Whitney *U*‐test (panel D, ***P = 0.0010) or unpaired *t*‐test with Welch's correction (panel E, ****P < 0.0001). Data are representative of two biological replicates. (F, G) Western blots showing biotin signal (F) and PD‐L1 and CMTM6 staining (G) in BLM WT cells after PD‐L1 surface proximity biotinylation and streptavidin bead pulldown, representative for *n* = 2 biological replicates. Molecular weight marker is in kDa. (H) Volcano plot showing enrichment and significance of proteins in BLM WT over BLM PD‐L1 KO after PD‐L1 surface proximity biotinylation and pull‐down followed by mass spectrometry. Dashed lines indicate cut‐off for significance (*P* < 0.001) and enrichment (> 6‐fold).

To investigate the PD‐L1 surface interactome in the human melanoma cell line BLM, we employed cell surface proximity biotinylation using an antibody targeting an extracellular epitope of endogenous PD‐L1 coupled to protein G‐horseradish peroxidase (HRP) (Fig. 1A). As a negative control, PD‐L1 knockout (KO) cells were generated using CRISPR‐Cas9 technology. Flow cytometric analysis confirmed the efficiency of the PD‐L1 KO strategy and the specificity of the PD‐L1 antibody, showing no detectable PD‐L1 expression in PD‐L1 KO cells as compared to wild‐type (WT) cells (Fig. 1B and Fig. S1A). Proximity biotinylation at the plasma membrane was analyzed by immunofluorescence (IF) showing the presence of biotin at the cell surface of WT cells, which was absent in PD‐L1 KO cells or controls omitting the biotinylation reaction (Fig. 1C,D and Fig. S1B,C). Quantification of biotin and PD‐L1 immunostaining by Pearson correlation coefficient analysis demonstrated positive colocalization of biotin with PD‐L1 (Fig. 1E), supporting the specificity of the proximity biotinylation assay. Pull down of biotinylated proteins with streptavidin beads in cell lysates showed enrichment of a broad range of biotinylated proteins and PD‐L1 in WT cells as compared to PD‐L1 KO cells (Fig. 1F,G). The known PD‐L1 binding protein CMTM6 [15, 19] was detected by western blot analysis in WT cells and not in PD‐L1 KO cells (Fig. 1G), further validating our approach.

Next, we performed mass spectrometry analysis on biotin‐labeled proteins captured by streptavidin beads. In total, 157 proteins were significantly enriched in the proximity biotinylation samples of WT cells compared to PD‐L1 KO cells (Table S1). Notably, the top 50 most significantly enriched proteins in the WT samples predominantly localized to the plasma membrane, validating the cell surface proximity biotinylation approach (Fig. S1D, Tables S2 and S3). These included the primary target of the assay PD‐L1 (CD274) and previously reported interaction partners integrin beta 4 (ITGB4) [42], CD58 (an established interactor of CMTM6) [43, 44] and HLA‐C, which was recently shown to be in close proximity to PD‐L1 [45] (Fig. 1H). Interestingly, the proteomic dataset revealed Tetraspanin‐4 (TSPAN4) as one of the most enriched proteins in PD‐L1 pull‐down samples of WT cells, representing a novel candidate interacting protein of PD‐L1. An independent PD‐L1 proximity biotinylation experiment followed by mass spectrometry analysis confirmed that TSPAN4 was significantly enriched in the pull‐down compared to an isotype control antibody (Fig. S2).

### 3.2 TSPAN4 colocalizes and interacts with PD‐L1 at the plasma membrane

**Fig. 2:**
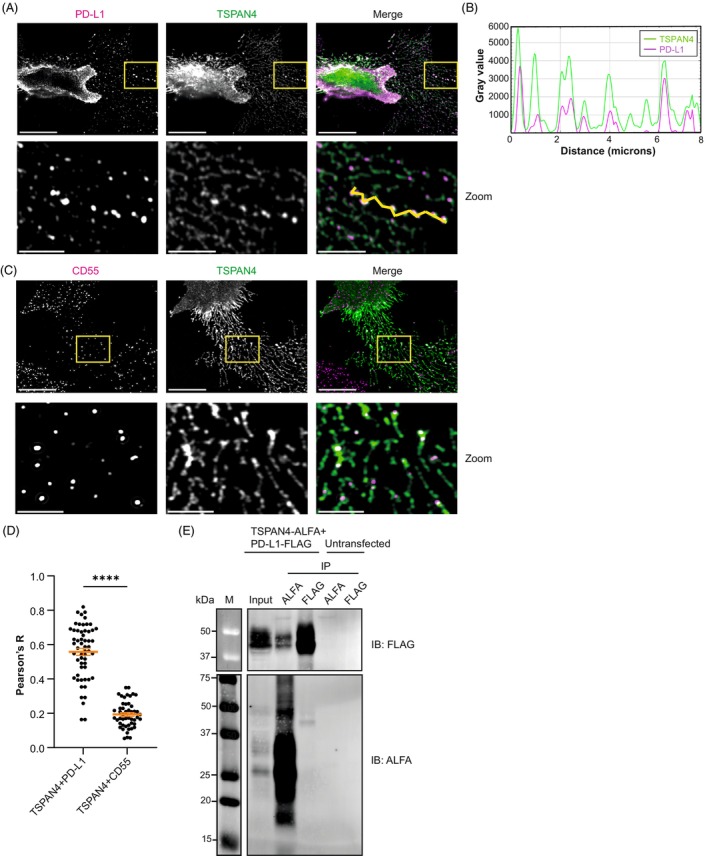
TSPAN4 colocalizes and interacts with PD‐L1 at the plasma membrane. (A, C) BLM WT cells were transfected with PD‐L1‐FLAG and TSPAN4‐ALFA and fixed on coverslips. (A) IF images of cells stained for PD‐L1‐FLAG and TSPAN4‐ALFA, with a merge of both channels, representative of 57 cells from *n* = 3 biological replicates. Lower panels are zoomed‐in images based on the box in the upper panels. For upper panels, scale bar: 10 μm. For lower panels, scale bar: 3 μm. (B) Profile plot of TSPAN4‐ALFA and PD‐L1‐FLAG from line drawn in (A). (C) IF images of cells stained for CD55 and TSPAN4‐ALFA, with a merge of both channels, representative of 49 cells from *n* = 3 biological replicates. Lower panels are zoomed‐in images based on the box in the upper panels. For upper panels, scale bar: 10 μm. For lower panels, scale bar: 3 μm. (D) Pearson correlation coefficient between TSPAN4‐ALFA and PD‐L1‐FLAG or CD55 (57 and 49 cells, respectively, from *n* = 3 biological replicates). Data are shown as mean ± SEM. Significance was determined by Mann–Whitney *U*‐test (****P < 0.0001). (E) Western blot showing PD‐L1‐FLAG in co‐IP upon TSPAN4‐ALFA IP when overexpressing both constructs in BLM WT, representative of *n* = 4 biological replicates. Molecular weight marker is in kDa.

Based on the proximity biotinylation results, we investigated whether TSPAN4 resided in close proximity to PD‐L1 in the cell. To this end, we overexpressed ALFA‐tagged TSPAN4 (TSPAN4‐ALFA) and FLAG‐tagged PD‐L1 (PD‐L1‐FLAG) to study their colocalization. Airyscan confocal microscopy showed the clear presence of retraction fibers and migrasomes (Fig. 2A), where TSPAN4 localization has been reported [46, 47, 48]. PD‐L1 and TSPAN4 were distributed along the plasma membrane and also localized to retraction fibers and migrasomes. We observed clear colocalization between PD‐L1‐FLAG and TSPAN4‐ALFA, which was most evident along retraction fibers and in migrasomes (Fig. 2A,B), whereas the membrane protein CD55 that served as a negative control showed no significant colocalization with TSPAN4‐ALFA (Fig. 2C,D). To further validate the interaction at the molecular level, we performed co‐immunoprecipitation (co‐IP) after transfection of TSPAN4‐ALFA and PD‐L1‐FLAG. Western blot analysis showed detection of PD‐L1‐FLAG upon immunoprecipitation of TSPAN4‐ALFA, which was not observed in untransfected cells (Fig. 2E and Fig. S6A). TSPAN4 [49] and PD‐L1 [50] are both glycosylated as indicated by their molecular weight of 25–35 and 45 kDa, respectively. These results indicate that PD‐L1 and TSPAN4 colocalize and interact on the cell surface.

### 3.3 TSPAN4 negatively regulates PD‐L1 expression at the cell surface

**Fig. 3:**
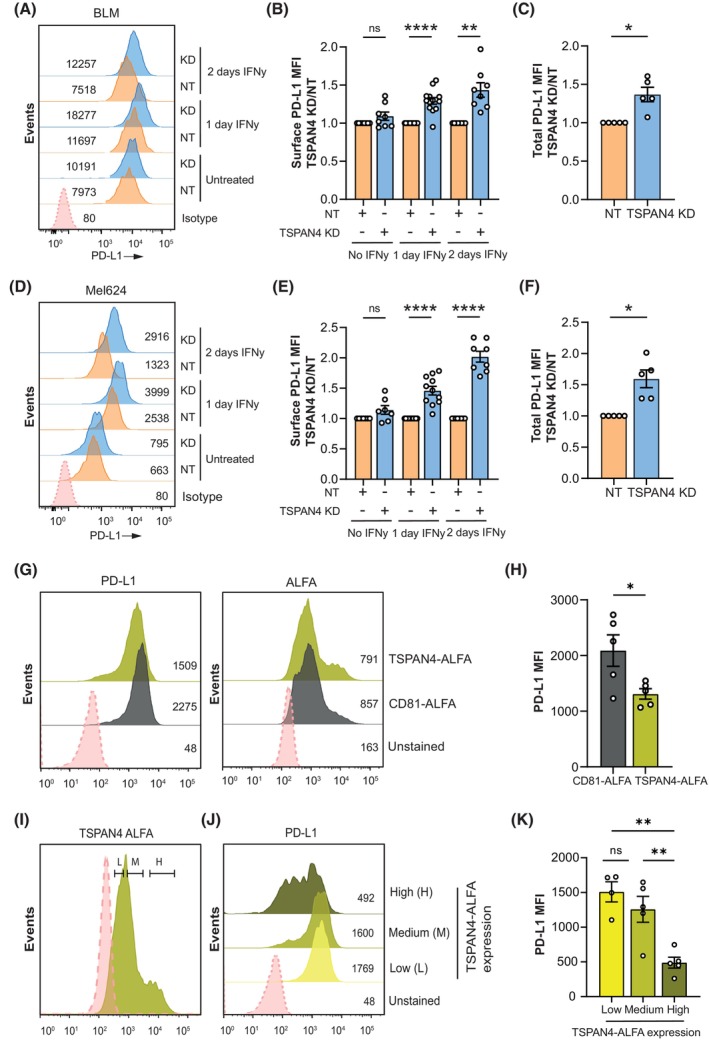
TSPAN4 negatively regulates PD‐L1 expression at the cell surface. (A, D) Representative flow cytometry PD‐L1 surface expression histograms of BLM (A) or Mel624 (D) NT and TSPAN4 KD cells treated with or without IFNγ for 1 or 2 days. (B, E) Flow cytometry surface PD‐L1 median fluorescence intensity (MFI) ratio of TSPAN4 KD over NT BLM (B) or Mel624 (E) cells treated with or without IFNγ. Significance was determined by one sample *t*‐test (panel B: ns, *P* = 0.1373, ****P < 0.0001, **P = 0.0025, panel E: ns, *P =* 0.0958, ****P < 0.0001). (C, F) Flow cytometry total (after permeabilization) PD‐L1 median fluorescence intensity ratio of TSPAN4 KD over NT BLM (C) or Mel624 (F) cells treated with IFNγ for 2 days. Significance was determined by one sample *t*‐test (panel C: *P = 0.0171, panel F: *P = 0.0134). (G) Flow cytometry PD‐L1 surface expression and ALFA expression histograms of Mel624 cells expressing TSPAN4‐ALFA or CD81‐ALFA. (H) Flow cytometry surface PD‐L1 median fluorescence intensity values of Mel624 cells overexpressing CD81‐ALFA or TSPAN4‐ALFA. Significance was determined by unpaired two‐tailed Student's *t*‐test (*P = 0.031). (I, J) Flow cytometry ALFA signal (I) or surface PD‐L1 signal (J) histograms of Mel624 cells overexpressing TSPAN4 ALFA, with Low, Medium, and High ALFA signal gating. (K) Flow cytometry surface PD‐L1 median fluorescence intensity values of Mel624 cells overexpressing TSPAN4‐ALFA, with Low, Medium, and High ALFA expression as in (J). Significance was determined by one‐way ANOVA with Tukey's multiple comparisons test (ns, *P* = 0.4731, L vs. H **P = 0.0013, L vs. M **P = 0.0064). (B, C, E, F, H, L) All dots represent biological replicates, and data are shown as mean ± SEM. Numbers of biological replicates are: panels B + E (surface PD‐L1, TSPAN4 KD vs. NT) – BLM: 0 day IFNγ *n* = 8; 1 day IFNγ *n* = 13; 2 days IFNγ *n* = 8; Mel624: 0 day IFNγ *n* = 7; 1 day IFNγ *n* = 11; 2 days IFNγ *n* = 8; Panels C + F (total PD‐L1, TSPAN4 KD vs. NT) – BLM *n* = 5; Mel624 *n* = 5; panel H (CD81‐ALFA vs. TSPAN4‐ALFA surface PD‐L1) *n* = 5; panel K (Low/Medium/High ALFA gating) *n* = 5.

Tetraspanins are known to regulate membrane proteins by altering their expression levels, mobility, and/or activity at the cell surface [23]. To study the effect of TSPAN4 on PD‐L1, we generated TSPAN4 knockdown (KD) in the melanoma cell lines BLM and Mel624 using an siRNA approach. Given that commercially available TSPAN4 antibodies were nonspecific in our hands, TSPAN4 KD efficiency was validated at the mRNA level (Fig. S3A). We observed about 80% reduction in *TSPAN4* mRNA levels 4–6 days after transfection. Next, we investigated the impact of TSPAN4 KD on PD‐L1 levels with and without interferon gamma (IFNγ) stimulation to induce transcriptional upregulation of *CD274/PD‐L1* [51]. BLM cells showed higher endogenous PD‐L1 protein expression compared to Mel624 as detected by flow cytometry (Fig. 3A,D). In both cell lines, PD‐L1 cell surface expression increased upon TSPAN4 KD, especially after longer IFNγ treatment (Fig. 3A,B,D,E and Fig. S3B). After 2‐day IFNγ stimulation, surface PD‐L1 was 1.4‐fold higher in BLM TSPAN4 KD cells, and 2‐fold higher in Mel624 TSPAN4 KD cells (Fig. 3B,E). In both BLM and Mel624 cell lines, total PD‐L1 levels also increased after TSPAN4 KD by 1.3‐ and 1.6‐fold, respectively (Fig. 3C,F). To study whether TSPAN4 altered PD‐L1 protein expression levels specifically, we investigated the effect of TSPAN4 KD on PD‐L2 and HLA‐ABC levels. HLA‐ABC expression was regulated by IFNγ stimulation in both cell lines, whereas PD‐L2 was only expressed in BLM cells (Fig. S3C,E). Importantly, both HLA‐ABC (Fig. S3F,G) and PD‐L2 (Fig. S3C,D) levels were not increased upon TSPAN4 KD, showing that TSPAN4 specifically affects PD‐L1 expression in melanoma cells.

Next, we investigated whether TSPAN4 overexpression would result in an opposite phenotype and reduce PD‐L1 expression levels. Ectopic overexpression of TSPAN4‐ALFA diminished endogenous PD‐L1 surface levels by about 35% in Mel624 cells as compared to a negative control (tetraspanin CD81‐ALFA) (Fig. 3G,H). Further analysis revealed a dose‐dependent effect of TSPAN4‐ALFA expression on the reduction of surface PD‐L1 levels (Fig. 3I–K). The BLM cell line with higher endogenous PD‐L1 levels revealed no significant effect on surface PD‐L1 levels upon overexpressing TSPAN4‐ALFA (Fig. S3H,I). Overall, these results indicate TSPAN4 negatively regulates PD‐L1 surface expression in melanoma cell lines.

### 3.4 TSPAN4 restricts the lateral mobility of PD‐L1 at the plasma membrane

**Fig. 4:**
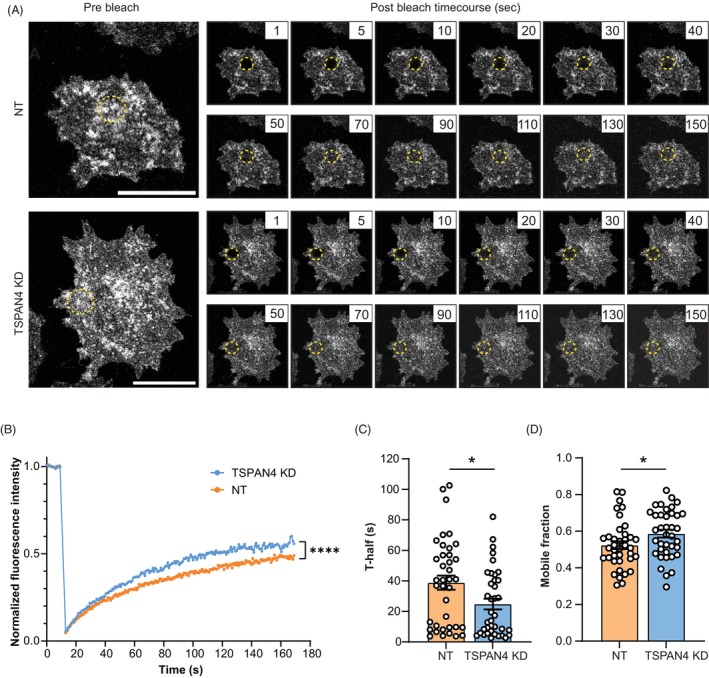
TSPAN4 restricts the lateral mobility of PD‐L1 at the plasma membrane. (A) BLM cells were stained with a fluorescent anti‐PD‐L1 antibody and FRAP was performed. Pre‐ and postbleach images of a PD‐L1 FRAP experiment with a BLM TSPAN4 KD and a NT cell. Cells are representative of 38 NT and 38 TSPAN4 KD cells from *n* = 3 biological replicates. Circle indicates area of bleaching and number indicates time after bleaching in seconds. Scale bar: 25 μm. (B) Normalized fluorescence intensity recovery curves consisting of 12 TSPAN4 KD and 12 NT BLM WT cells from one representative PD‐L1 FRAP experiment out of three biological replicates. Significance was determined by two‐tailed Wilcoxon matched‐pairs signed rank test (****P < 0.0001). (C, D) T‐half (C) and mobile fraction (D) derived from 38 NT and 38 TSPAN4 KD cells from *n* = 3 biological replicates. All dots represent individual cells. Data are shown as mean ± SEM. Significance was determined by two‐tailed Mann–Whitney *U*‐test (C) (*P = 0.0288) or two‐tailed unpaired *t*‐test with Welch's correction (D) (*P = 0.0387).

Tetraspanins are able to affect the lateral mobility of associated partner proteins at the plasma membrane [23, 52]. First, we assessed whether PD‐L1 molecules displayed lateral mobility at the cell surface of melanoma cells by performing Fluorescence Recovery After Photobleaching (FRAP) on endogenous PD‐L1 at the cell surface (Fig. 4A). This technique enables the quantification of the lateral mobility of plasma membrane proteins by measuring the extent and rate of fluorescence recovery over time following the photobleaching of a small region of the cell surface. After bleaching an area of 5 μm in diameter on the plasma membrane, the PD‐L1 signal was partially recovered (Fig. 4B), with a half‐time of recovery of approximately 40 s (Fig. 4C) and a mobile fraction of about 50% (Fig. 4D). This indicates that a proportion of cell surface PD‐L1 molecules is restricted in their lateral mobility. Interestingly, TSPAN4 KD resulted in a markedly faster PD‐L1 recovery (recovery half‐time of 25 versus 39 s, and about a 5–10% higher mobile fraction of PD‐L1). These data demonstrate that PD‐L1 displays lateral mobility at the cell surface of melanoma cells, which is restricted by TSPAN4.

### 3.5 TSPAN4 mediates PD‐L1 degradation by competing with CMTM6 binding to PD‐L1

**Fig. 5:**
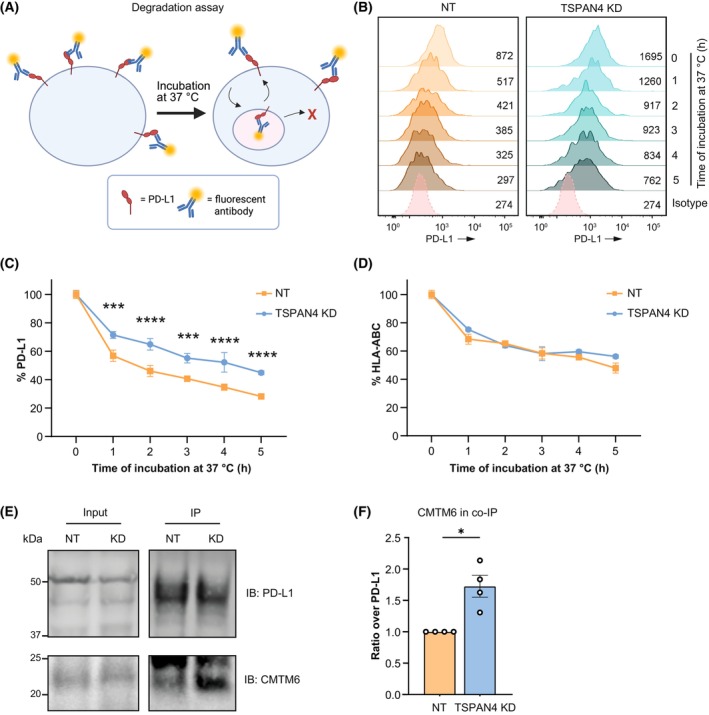
TSPAN4 mediates PD‐L1 degradation by competing with CMTM6 for binding to PD‐L1. (A) Schematic of flow cytometric‐based degradation assay. Cells are labeled with fluorescent antibodies, and loss of signal is measured after incubation at 37 °C. Created in BioRender. Franken, G. (2025) https://BioRender.com/no8getf. (B) Flow cytometry PD‐L1 surface expression histograms of Mel624 NT or TSPAN4 KD cells from degradation assay as in (A), representative of *n* = 3 biological replicates. (C, D) PD‐L1 (C) and HLA‐ABC (D) degradation plots. In (C, D), data are representative of *n* = 3 biological replicates and are shown as mean with range of *n* = 3 technical replicates. Significance was determined by ordinary two‐way ANOVA with Šidák's multiple comparison's test (***P = 0.0002, ****P < 0.0001). (E) Western blot showing PD‐L1 and CMTM6 signal from PD‐L1 IP in Mel624 TSPAN4 KD or NT cells, representative of *n* = 4 biological replicates. Molecular weight marker is in kDa. (F) Normalized CMTM6 signal in PD‐L1 IP taken as ratio over PD‐L1 in Mel624 TSPAN4 KD or NT cells. Each dot represents an individual biological replicate, and data are shown as mean ± SEM. Significance was determined by one sample *t*‐test (*P = 0.0253).

Endocytosis and subsequent recycling within the endosomal compartment, as well as lysosomal or proteasomal degradation, are mechanisms by which the homeostasis of cell surface proteins can be dynamically regulated [18]. To investigate the molecular mechanisms responsible for the increased surface expression of PD‐L1 in TSPAN4 KD cells, we measured the PD‐L1 degradation over time in Mel624 cells using a flow cytometric‐based degradation assay (Fig. 5A). In this assay, cell surface PD‐L1 was stained with a PD‐L1 antibody conjugated to a fluorophore, and degradation was measured over time by quantifying the decrease in fluorescent signal. This approach showed a steady decline of signal over time (Fig. 5B and Fig. S4A), with on average about 80% of signal reduction after 5 h incubation, consistent with degradation rates of PD‐L1 observed previously [15, 20]. Upon TSPAN4 KD, the degradation rate of PD‐L1 was delayed, with an average of 60% decrease after 5 h (Fig. 5C and Fig. S4C). In contrast, the degradation rate of control cell surface protein HLA‐ABC was not affected by TSPAN4 KD (Fig. 5D and Fig. S4B,C), indicating specificity of TSPAN4 for PD‐L1.

CMTM6, a known interaction partner of PD‐L1, enhances PD‐L1 surface levels by redirecting endocytosed PD‐L1 back towards the plasma membrane, preventing lysosomal degradation [15, 19]. We assessed whether TSPAN4 might affect the degree to which CMTM6 interacted with PD‐L1. Upon IP of PD‐L1, we could readily detect CMTM6 in association with PD‐L1 by western blotting, which was markedly enhanced by about 70% upon TSPAN4 KD (Fig. 5E,F and Figs S4B,C and S6B). These data indicate an increased association of PD‐L1 with CMTM6 in TSPAN4 KD cells, resulting in a slower degradation rate and increased PD‐L1 levels. In BLM cells, the association between CMTM6 and PD‐L1 was not affected after TSPAN4 KD (Figs S4D–G and S6C).

### 3.6 TSPAN4 impacts PD‐1 binding and T‐cell activation

**Fig. 6:**
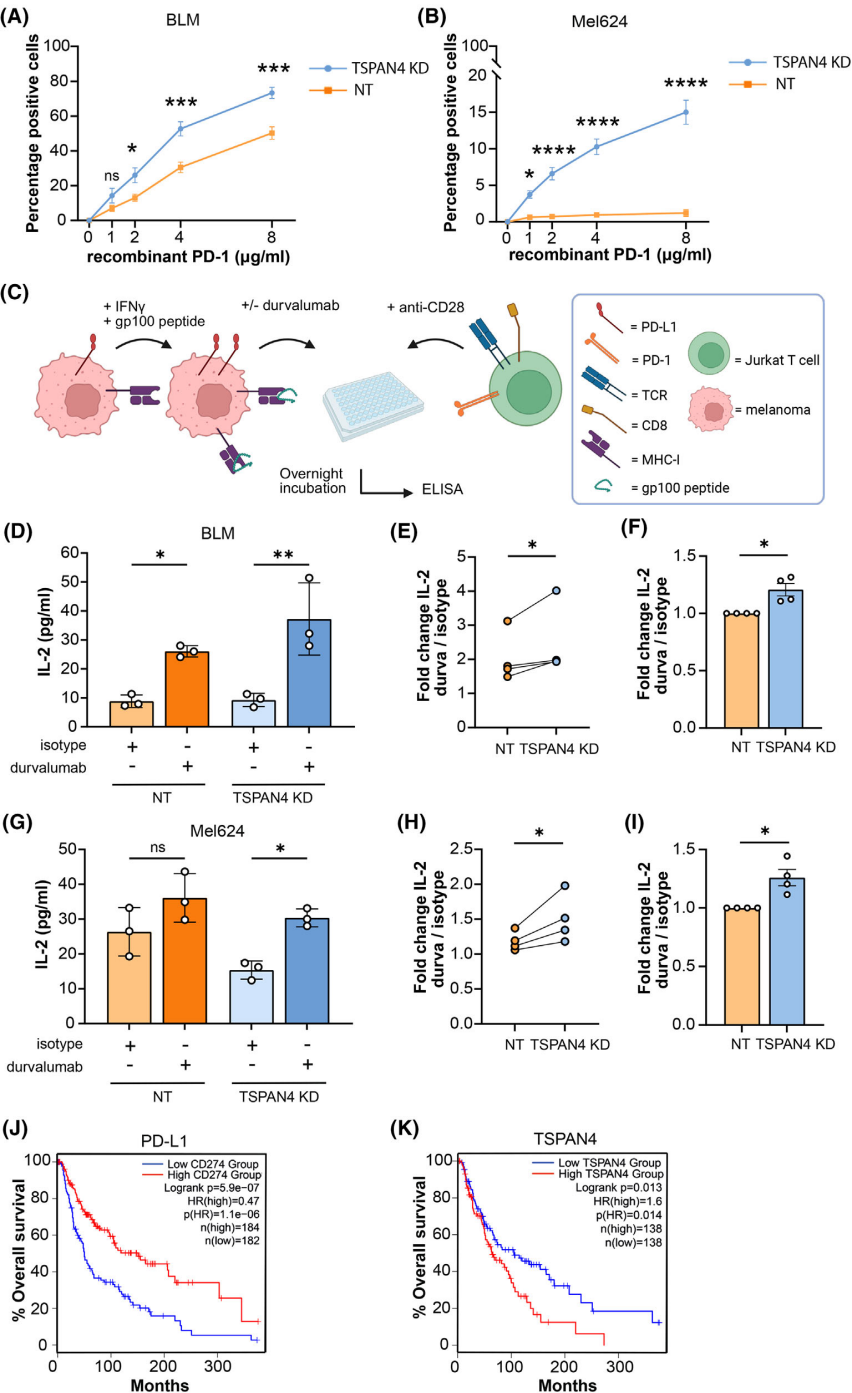
TSPAN4 impacts PD‐1 binding and T‐cell activation. (A, B) Cells were incubated with recombinant PD‐1 at different concentrations, and PD‐1 binding was measured by flow cytometry. Percentage PD‐1+ cells after recombinant PD‐1 treatment in BLM (A) or Mel624 (B) TSPAN4 KD or NT cells. Data are from *n* = 3 biological replicates shown as mean ± SEM. Significance was determined by ordinary two‐way ANOVA with Šidák's multiple comparison's test (panel A: ns, *P* > 0.9999, ns, *P =* 0.4303, *P = 0.0355, ***P = 0.0002, ***P = 0.0001, panel B: ns, *P* > 0.9999, *P = 0.030, ****P < 0.0001). (C) Schematic of Jurkat coculture assay. Melanoma cell lines are gp100 peptide‐pulsed and treated with or without durvalumab, and co‐cultured with Jurkat T cells. After overnight incubation, supernatant is taken for ELISA. Created in BioRender. Franken, G. (2025) https://BioRender.com/wi89t1a. (D, G) IL‐2 ELISA experiment results from Jurkat T‐cell coculture assay with BLM (D) or Mel624 (G) TSPAN4 KD and NT cells, representative of *n* = 4 biological replicates. Dots are from *n* = 3 technical replicates and are shown as mean ± SD. Significance was determined by ordinary one‐way ANOVA with Tukey's multiple comparisons test (panel D: *P = 0.0476, **P = 0.0034, panel G: ns, *P* = 0.1828, *P = 0.0328). (E, H) IL‐2 fold change of durvalumab‐treated over isotype‐treated samples in TSPAN4 KD and NT BLM (E) or Mel624 (H) cells. Dots are from *n* = 4 biological replicates and conditions belonging to the same experiment are connected with a line. Significance was determined by paired one‐tailed Student's *t*‐test (panel E: *P = 0.0385, panel H: *P = 0.0275). (F, I) IL‐2 fold change of durvalumab‐treated over isotype‐treated samples in TSPAN4 KD and NT BLM (F) or Mel624 (I) cells, normalized to the NT condition. Dots are from *n* = 4 biological replicates, and data is shown as mean ± SEM. Significance was determined by one sample *t*‐test (panel F: *P = 0.0313, panel I: *P = 0.0335). (J, K) Overall survival in patients with skin cutaneous melanoma with high or low mRNA expression levels of PD‐L1 (J) and TSPAN4 (K).

PD‐L1 expression levels on tumor cells are implicated as a prognostic factor of sensitivity to PD‐L1 antibody blockade therapy [8]. To investigate the functional effect of increased PD‐L1 levels in TSPAN4 KD cells, we tested PD‐1 binding by using soluble recombinant PD‐1 protein. We observed that PD‐1 binding was enhanced upon TSPAN4 KD in both BLM and Mel624 cells (Fig. 6A,B and Fig. S5A,B). Next, T‐cell responses were measured in a Jurkat T‐cell line model stably expressing a gp100‐specific TCR, as well as CD8 [38], and PD‐1 (Fig. S5C,D). These Jurkat T cells were cocultured with gp100 peptide‐pulsed melanoma cells, and T‐cell activation was analyzed by measuring IL‐2 production in supernatants after 24 h by ELISA (Fig. 6C). We established whether this model system was dependent on PD‐L1 and PD‐L2 for the activation of T‐cell responses, as both proteins can inhibit T cells by binding PD‐1 [51]. Jurkat T cells were cocultured with WT, PD‐L1 KO, and PD‐L2 KO BLM cell lines, and Jurkat T‐cell activation was determined by IL‐2 production (Fig. S5E). Durvalumab treatment enhanced IL‐2 production by Jurkat T cells with BLM WT and BLM PD‐L2 KO cells by about 50%, but not with BLM PD‐L1 KO cells. These data demonstrate that PD‐L1 and PD‐L2 can both inhibit Jurkat T cells through PD‐1 binding; however, durvalumab specifically prevents signaling mediated by PD‐L1. Subsequently, we investigated whether increased levels of PD‐L1 in TSPAN4 KD melanoma cells showed an impact on the ability of durvalumab to activate T‐cell responses. Durvalumab enhanced IL‐2 production in Jurkat T cells incubated with peptide‐loaded melanoma cells (Fig. 6D,G). However, durvalumab had a significantly larger effect on IL‐2 production upon TSPAN4 KD, indicating a stronger potential of durvalumab to activate T cells in the absence of TSPAN4 (Fig. 6D–I). TSPAN4 KD led to increased surface expression of PD‐L1, which enhances its engagement with PD‐1 on Jurkat T cells (Fig. 6A,B). Consequently, blocking this interaction with durvalumab resulted in a greater relief of PD‐1‐mediated inhibition in TSPAN4 KD cells.

Finally, we analyzed the overall survival of skin cutaneous melanoma patients in relation to *PD‐L1* and *TSPAN4* mRNA expression based on publicly available datasets. These data show that patients with higher levels of PD‐L1 expression on melanoma display a better overall survival compared to patients with lower levels of PD‐L1 on the tumor (Fig. 6J). On the contrary, *TSPAN4* expression was inversely correlated with the overall survival of melanoma patients (Fig. 6K), in line with the negative regulation of TSPAN4 on PD‐L1 levels. Taken together, our data demonstrate that TSPAN4 impacts PD‐1 binding and sensitivity to durvalumab, and TSPAN4 is related to the clinical outcome of melanoma patients. This suggests that increasing PD‐L1 levels in melanoma cells by targeting TSPAN4 may improve the therapeutic effect of immune checkpoint inhibitors like durvalumab.

## 4 Discussion

PD‐L1 is a promising target in immunotherapy for a broad range of cancer types, and PD‐L1/PD‐1 checkpoint blockade therapy is the standard of care in metastatic melanoma [7, 8]. Yet, treatment responses are limited and the molecular regulation of PD‐L1 is incompletely understood. Here, we present the cell surface interactome of endogenous PD‐L1 using proximity biotinylation followed by mass spectrometry analysis. This approach has the advantage that the PD‐L1 native environment is kept intact, in contrast to conventional immunoprecipitation or epitope‐tagged PD‐L1 studies [45, 53] that may interfere with its normal physiological function.

We identified TSPAN4 as a novel PD‐L1 interaction partner, whereby TSPAN4 and PD‐L1 colocalize in retraction fibers and migrasomes. Migrasomes are vesicles originating from retraction fibers when cells migrate over the extracellular matrix in a complex series of biophysical events. Migrasomes play a role in homeostasis maintenance, cell‐to‐cell communication, and metastasis by releasing chemokines [54, 55, 56]. PD‐L1 has previously been shown to reside at the cell rear and migrasomes in breast carcinoma cells [42], which is similar to the described localization of TSPAN4 [46, 47, 48]. It is interesting to contemplate whether both proteins together may exert synergistic effects on cell proliferation, migration, and migrasome formation.

PD‐L1 undergoes constitutive internalization via a clathrin‐ and dynamin‐dependent pathway, independent of PD‐1 engagement [57], and is recycled back to the cell surface by interaction with CMTM and TRAPPC family members [15, 58]. Based on our findings showing that TSPAN4 KD enhanced the interaction of PD‐L1 with CMTM6, we propose a model whereby TSPAN4 prevents PD‐L1 association with CMTM6, thereby promoting PD‐L1 degradation upon internalization. PD‐L2 expression was not affected by the absence of TSPAN4, indicating a specific modulation of only one of the PD‐1 ligands by TSPAN4. Likewise, HLA‐ABC expression was not affected by TSPAN4 KD in BLM and Mel624 cells. This is in line with previous findings showing that CMTM6 did not affect PD‐L2 or HLA‐ABC expression levels [19].

TSPAN4 KD resulted in higher PD‐L1 expression levels in both melanoma cell lines, yet the underlying mechanism may differ. TSPAN4 KD in BLM cells did not result in an increased association of PD‐L1 with CMTM6 which may be related to the 6‐ to 10‐fold higher PD‐L1 levels and slower PD‐L1 degradation rate in BLM cells compared to Mel624 cells. Tetraspanins are able to regulate protein trafficking at multiple stages, including transportation from the endoplasmic reticulum and Golgi towards the plasma membrane [59]. Notable examples are CD81 regulating CD19 trafficking in B cells [60, 61], and TSPANC8 proteins regulating transmembrane metalloprotease ADAM10 trafficking in HeLa cells [62]. TSPAN4 may regulate the trafficking of newly synthesized PD‐L1 towards the plasma membrane. Interestingly, a negative correlation between *TSPAN7* and *PD‐L1* mRNA expression has been observed in glioma [63], indicating a possible redundancy with other tetraspanins. There are other examples of tetraspanins attenuating surface expression of interacting partner proteins by enhancing endocytosis, including TSPAN5 regulating ADAM10 [64], and CD82 with ligand‐bound EGFR [65, 66]. Furthermore, a subset of tetraspanins contains tyrosine‐ or dileucine‐based sorting sequences at their C‐terminal domains, which may promote internalization of these tetraspanins and partner proteins [67]. TSPAN4 also contains a tyrosine‐based sorting sequence, but it remains to be established whether this motif is functional and whether it is involved in regulating PD‐L1 internalization and intracellular trafficking.

We demonstrate that PD‐L1 is present in two different populations: a mobile and immobile fraction. TSPAN4 KD increased both the speed of and the mobile fraction of PD‐L1 molecules indicating that TSPAN4 arrests PD‐L1 on the cell surface. Tetraspanins have been reported to affect lateral mobility of their interacting proteins, like tetraspanin CD81 that restricts the mobility of CD19 [52]. The lateral mobility of membrane proteins is very important in dynamic cell processes, such as immunological synapse (IS) formation between T cells and antigen‐presenting cells [22, 25]. This is a highly spatiotemporally regulated event, with segregation of relevant molecules in concentric domains. PD‐L1 on antigen‐presenting cells is also recruited to the IS, which coincides with the formation of PD‐1 microdomains on the T‐cell side that are important for T‐cell inhibition [5, 6, 22]. Since IS formation is a spatiotemporal process that relies on orderly and timely recruitment of surface proteins, it would be interesting to investigate the effect of PD‐L1 mobility in IS formation in the presence and absence of TSPAN4.

We observed TSPAN4 KD cells have enhanced binding capacity to PD‐1 resulting in more efficient immune checkpoint blockade in T cells. This effect is most likely mediated by increased surface PD‐L1 levels in TSPAN4 KD cells, which was most apparent in Mel624, where basal PD‐L1 levels are relatively low. Several studies have indicated that patients with high PD‐L1 levels are more sensitive to immune checkpoint blockade therapy and show a better overall survival in melanoma [68] glioma [69], and non‐small‐cell lung cancer [70]. Similarly, our *PD‐L1* patient cohort shows a correlation between high expression and better overall survival. An open question is whether TSPAN4 expression on melanoma can predict response to PD‐L1 checkpoint blockade therapy. Unfortunately, this question is difficult to address with the TCGA‐SKCM cohort, as complete systematic treatment annotation is not available [40]. TSPAN4 is known to promote cell proliferation, migration, and migrasome formation [47, 48, 49], all of which are pro‐tumorigenic. Our data indicate that TSPAN4 may additionally negatively affect survival by lowering PD‐L1 levels, making PD‐L1 antibody therapy less effective. Characterization of the tumor‐microenvironment in the presence and absence of TSPAN4 and the efficacy of PD‐L1 antibody therapy could shed light on this. Since TSPAN4 is expressed ubiquitously, with phenotypic effects in, for example, kidney carcinoma [48], it is possible that a functional PD‐L1‐TSPAN4 interaction is not limited to melanoma.

## 5 Conclusion

In conclusion, this study shows that TSPAN4 controls PD‐L1 membrane expression, mobility, and degradation rate through a newly defined regulatory axis. The absence of TSPAN4 sensitizes melanoma cells to anti‐PD‐L1 immune checkpoint therapy, providing a rationale for targeting TSPAN4 to enhance treatment outcome in melanoma patients.

## Conflict of interest

The authors declare no conflict of interest.

## Author contributions

GAF: conceptualization; formal analysis; investigation; visualization; methodology; writing – original draft; writing – review and editing. AAG: investigation, formal analysis, and visualization. CGS: data curation and formal processing. MV: data curation. IR: investigation and visualization. GM: data curation; formal analysis; and visualization. BS: supervision; writing – review and editing. ABS: conceptualization; supervision; methodology; writing – review and editing; funding acquisition; and project administration.

## Supporting information


**Fig. S1.** Identification of PD‐L1 interacting proteins in melanoma by proximity biotinylation followed by mass spectrometry.
**Fig. S2.** Identification of PD‐L1 interacting proteins in melanoma by proximity biotinylation followed by mass spectrometry.
**Fig. S3.** TSPAN4 negatively regulates PD‐L1 expression at the cell surface.
**Fig. S4.** TSPAN4 mediates PD‐L1 degradation by competing with CMTM6 binding to PD‐L1.
**Fig. S5.** TSPAN4 impacts PD‐1 binding and T cell activation.
**Fig. S6.** Raw western blot membranes.
**Table S1.** Mass‐spectrometry data of the PD‐L1 proximity biotinylation assay in BLM WT vs BLM PD‐L1 KO cells, with all differential hits of WT vs PD‐L1 KO cells arranged by fold difference (log_2_), with significance indicated.
**Table S2.** The top 50 significantly enriched hits in WT cells from the mass‐spectrometry data of the PD‐L1 proximity biotinylation assay that were taken for GO analysis.
**Table S3.** GO analysis of the top 50 significantly enriched hits in WT cells from the mass‐spectrometry data of the PD‐L1 proximity biotinylation assay, with false discovery rate, cellular compartment, and proteins assigned to these cellular compartments indicated.

## Data Availability

The mass spectrometry proteomics data have been deposited to the ProteomeXchange Consortium via the PRIDE [70] partner repository with the dataset identifier PXD064698. Token: dfEK8v4Gtjna.
