# Supplementary material for: Cell surface interactome analysis identifies TSPAN4 as a negative regulator of PD‐L1 in melanoma
Source: Mol Oncol. 2026 Jan 12;20(5):1140–60. doi: 10.1002/1878-0261.70182 (PMC13155135; doi:10.1002/1878-0261.70182)
Supplement: Supplementary file 1 — Fig. S1. Identification of PD‐L1 interacting proteins in melanoma by proximity biotinylation followed by mass spectrometry. Fig. S2. Identification of PD‐L1 interacting proteins in melanoma by proximity biotinylation followed by mass spectrometry. Fig. S3. TSPAN4 negatively regulates PD‐L1 expression at the cell surface. Fig. S4. TSPAN4 mediates PD‐L1 degradation by competing with CMTM6 binding to PD‐L1. Fig. S5. TSPAN4 impacts PD‐1 binding and T cell activation. Fig. S6. Raw western blot membranes. Table S1. Mass‐spectrometry data of the PD‐L1 proximity biotinylation assay in BLM WT vs BLM PD‐L1 KO cells, with all differential hits of WT vs PD‐L1 KO cells arranged by fold difference (log2), with significance indicated. Table S2. The top 50 significantly enriched hits in WT cells from the mass‐spectrometry data of the PD‐L1 proximity biotinylation assay that were taken for GO analysis. Table S3. GO analysis of the top 50 significantly enriched hits in WT cells from the mass‐spectrometry data of the PD‐L1 proximity biotinylation assay, with false discovery rate, cellular compartment, and proteins assigned to these cellular compartments indicated. [file MOL2-20-1140-s001.zip › Supporting_information_legends.docx]

**Supporting information legends**

**Figure S1. Identification of PD-L1 interacting proteins in melanoma by proximity biotinylation followed by mass spectrometry**

(**A**) Flow cytometry surface PD-L1 median fluorescence intensity (MFI) values for WT and PD-L1 KO BLM cells after 1-day IFNγ treatment. Data is from n=3 biological replicates. (**B**) Representative IF overview of BLM WT (upper panels) and PD-L1 KO (lower panels) cells stained for PD-L1, biotin, and DAPI after PD-L1 surface proximity biotinylation for two biological replicates. Scale bar: 50 µm. (**C**) Representative IF overview of BLM WT cells stained for PD-L1, biotin, and DAPI after PD-L1 surface proximity biotinylation protocol without H₂O₂ (upper panels) or without antibody proteinG-HRP incubation (lower panels) for two biological replicates. Scale bar: 50 µm. (**C**) Predominant subcellular localization of top 50 significantly WT-enriched proteins from the mass-spectrometry data of the PD-L1 proximity biotinylation assay, with false discovery rate indicated. Analysis performed by GO analysis using SubCellularVis [41].

**Figure S2. Identification of PD-L1 interacting proteins in melanoma by proximity biotinylation followed by mass spectrometry**

Volcano plot showing enrichment and significance of proteins in BLM WT cells after PD-L1 surface proximity biotinylation and pull-down followed by mass spectrometry, with a mouse IgG1 isotype control. Dashed lines indicate cut-off for significance (p<0.01) and enrichment (>2-fold).

**Figure S3. TSPAN4 negatively regulates PD-L1 expression at the cell surface**

(**A**) *TSPAN4* mRNA levels of *TSPAN4* siRNA-transfected BLM or Mel624 cells as determined by qPCR, normalized to NT siRNA-transfected conditions, with data from n=12 and n=10 biological replicates for BLM and Mel624 respectively. Data is shown as mean +/- SEM. (**B**) Gating strategy of flow cytometry data, showing a representative example of BLM and Mel624 TSPAN4 KD and NT cells from n=13 and n=11 biological replicates respectively. Shown is gating on all cells, then single cells, then live cells. (**C,E**) flow cytometry PD-L2 surface expression histograms of BLM (C) or Mel624 (E) NT and TSPAN4 KD cells treated for 1 day with IFNγ, representative of n=3 biological replicates for each cell line. (**D**) Flow cytometry surface PD-L2 median fluorescence intensity ratio of TSPAN4 KD over NT BLM cells treated for 1 day with IFNγ, with data from n=3 biological replicates. Significance was determined by one sample t test (ns, *P*=0.7631). (**F,G**) Flow cytometry surface HLA-ABC median fluorescence intensity ratio of TSPAN4 KD over NT BLM (F) or Mel624 (G) cells treated for 1 or 2 days with IFNγ. Numbers of biological replicates are: BLM 1 day IFNγ n=3; 2 days IFNγ n=4; Mel624 1 day IFNγ n=3; 2 days IFNγ n=4. (**H**) Flow cytometry PD-L1 surface expression and ALFA signal histograms of BLM cells expressing CD81-ALFA or TSPAN4-ALFA, representative of n=5 biological replicates. (**I**) Flow cytometry surface PD-L1 median fluorescence intensity values of BLM cells overexpressing TSPAN4-ALFA and CD81-ALFA. Significance was determined by unpaired two tailed Student’s t test (ns, *P*=0.6455), with n=5 biological replicates. (A,D,F,G,I) Each dot represents an individual biological replicate, and data is shown as mean +/- SEM.

**Figure S4. TSPAN4 mediates PD-L1 degradation by competing with CMTM6 binding to PD-L1**

(**A**) Gating strategy of flow cytometry data from degradation assay of Mel624 TSPAN4 KD and NT cells, 0 hours or 5 hours after incubation at 37 °C, representative of n=3 biological replicates. Shown is gating on all cells, then single cells, then live cells. (**B**) Flow cytometry HLA-ABC surface expression histograms of Mel624 NT or TSPAN4 KD cells from degradation assay, representative of n=3 biological replicates. (**C**) Biological replicates of degradation assay for PD-L1 (left panels) or HLA-ABC (right panels) in Mel624 TSPAN4 KD and NT cells. Data are shown as mean with SD of n=2 (replicate 2) or n=3 (replicate 3) technical replicates. (**D,E**) Quantification of CMTM6 (D) and PD-L1 (E) in PD-L1 IP for TSPAN4 KD Mel624 cells normalized to the NT condition, with data from n=4 biological replicates. (**F**) Western blotshowing PD-L1 and CMTM6 signal from PD-L1 IP in BLM TSPAN4 KD and NT cells, representative of n=5 biological replicates. Molecular weight marker is in kDa. (**G,H,I**) Quantification of CMTM6 (G), PD-L1 (H), or CMTM6 over PD-L1 (I) in PD-L1 IP for TSPAN4 KD BLM cells normalized to the NT condition, with data from n=5 biological replicates. (D,E,G,H,I) Each dot represents an individual biological replicate, and data is shown as mean +/- SEM. Significance was determined by one sample t test (ns, *P*=0.7196).

**Figure S5. TSPAN4 impacts PD-1 binding and T cell activation**

(**A,B**) Flow cytometry recombinant PD-1 binding histograms of BLM (A) or Mel624 (B) NT and TSPAN4 KD cells treated for 2 days with IFNγ, representative of n=3 biological replicates. (**C,D**) Flow cytometry histograms of PD-1 (C) or CD8 (D) expression in Jurkat T cells used in the Jurkat co-culture assay, representative of n=3 biological replicates. (**E**) IL-2 ELISA experiment results from Jurkat co-culture assay with BLM WT, PD-L1 KO, or PD-L2 KO cells, gp100-peptide pulsed or not, treated with or without durvalumab, representative of n=4 biological replicates, dots indicate technical replicates, and are shown as mean +/- SD. Significance was determined by ordinary one-way ANOVA with Tukey’s multiple comparisons test (ns, *P*=0.8031, ***P*=0.0030, *****P*<0.0001).
