# Supplementary figures and images for: Cell surface interactome analysis identifies TSPAN4 as a negative regulator of PD‐L1 in melanoma
Source: Mol Oncol. 2026 Jan 12;20(5):1140–60. doi: 10.1002/1878-0261.70182 (PMC13155135; doi:10.1002/1878-0261.70182)

Figure S1

A

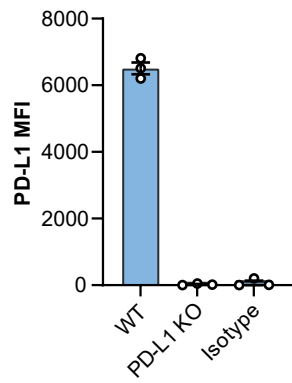

B

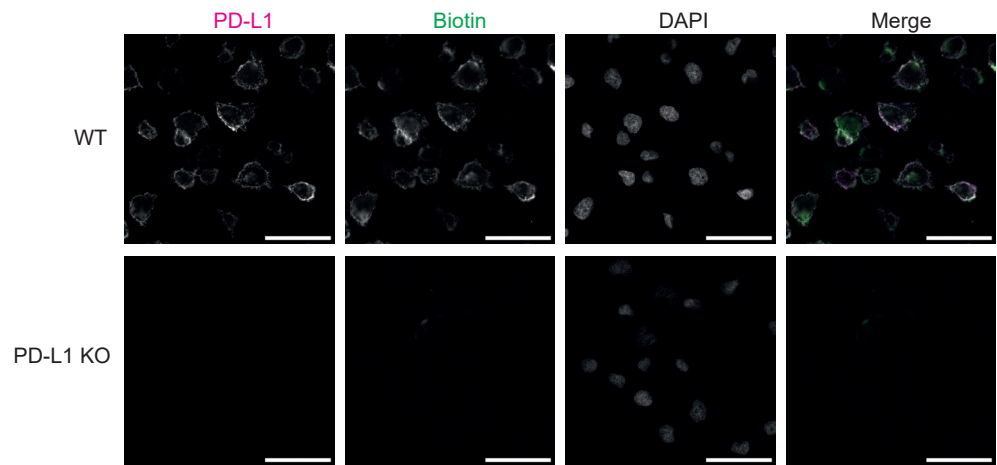

C

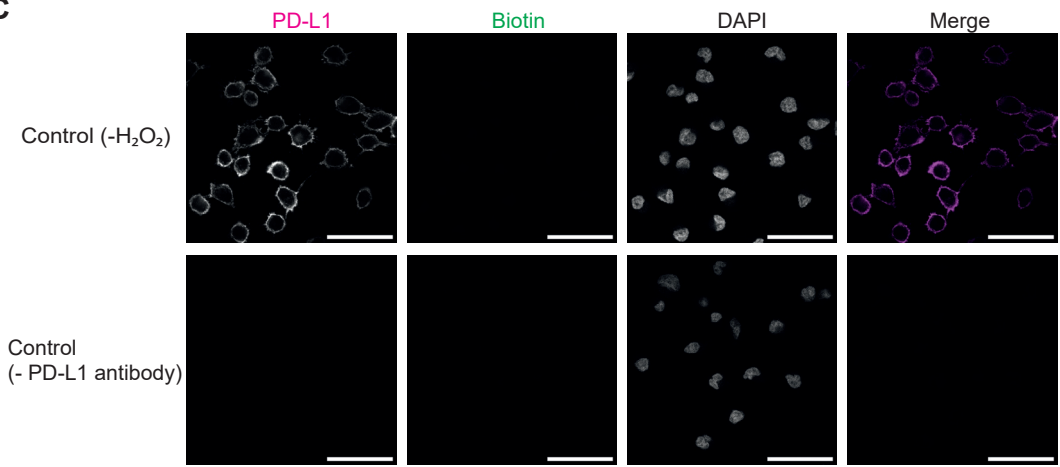

D

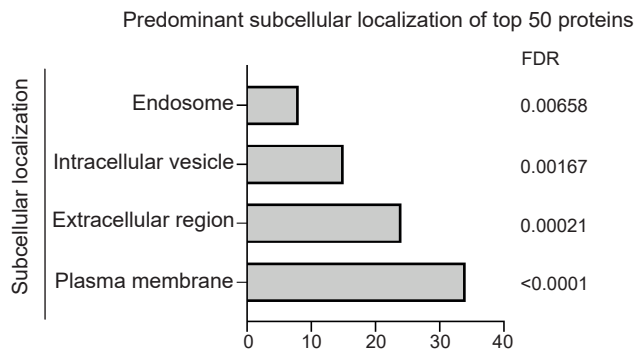

Supplement: Supplementary file 1 — Fig. S1. Identification of PD‐L1 interacting proteins in melanoma by proximity biotinylation followed by mass spectrometry. Fig. S2. Identification of PD‐L1 interacting proteins in melanoma by proximity biotinylation followed by mass spectrometry. Fig. S3. TSPAN4 negatively regulates PD‐L1 expression at the cell surface. Fig. S4. TSPAN4 mediates PD‐L1 degradation by competing with CMTM6 binding to PD‐L1. Fig. S5. TSPAN4 impacts PD‐1 binding and T cell activation. Fig. S6. Raw western blot membranes. Table S1. Mass‐spectrometry data of the PD‐L1 proximity biotinylation assay in BLM WT vs BLM PD‐L1 KO cells, with all differential hits of WT vs PD‐L1 KO cells arranged by fold difference (log2), with significance indicated. Table S2. The top 50 significantly enriched hits in WT cells from the mass‐spectrometry data of the PD‐L1 proximity biotinylation assay that were taken for GO analysis. Table S3. GO analysis of the top 50 significantly enriched hits in WT cells from the mass‐spectrometry data of the PD‐L1 proximity biotinylation assay, with false discovery rate, cellular compartment, and proteins assigned to these cellular compartments indicated. [file MOL2-20-1140-s001.zip › mol270182-sup-0001-FigureS1.pdf]

Figure S2

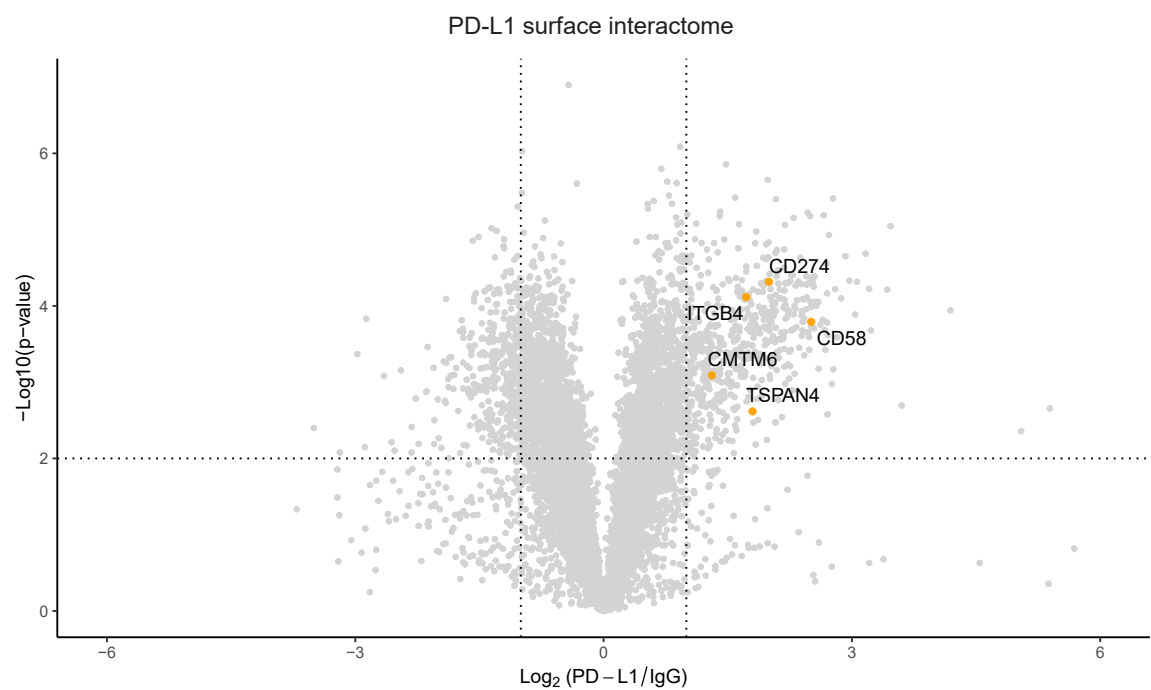

Supplement: Supplementary file 1 — Fig. S1. Identification of PD‐L1 interacting proteins in melanoma by proximity biotinylation followed by mass spectrometry. Fig. S2. Identification of PD‐L1 interacting proteins in melanoma by proximity biotinylation followed by mass spectrometry. Fig. S3. TSPAN4 negatively regulates PD‐L1 expression at the cell surface. Fig. S4. TSPAN4 mediates PD‐L1 degradation by competing with CMTM6 binding to PD‐L1. Fig. S5. TSPAN4 impacts PD‐1 binding and T cell activation. Fig. S6. Raw western blot membranes. Table S1. Mass‐spectrometry data of the PD‐L1 proximity biotinylation assay in BLM WT vs BLM PD‐L1 KO cells, with all differential hits of WT vs PD‐L1 KO cells arranged by fold difference (log2), with significance indicated. Table S2. The top 50 significantly enriched hits in WT cells from the mass‐spectrometry data of the PD‐L1 proximity biotinylation assay that were taken for GO analysis. Table S3. GO analysis of the top 50 significantly enriched hits in WT cells from the mass‐spectrometry data of the PD‐L1 proximity biotinylation assay, with false discovery rate, cellular compartment, and proteins assigned to these cellular compartments indicated. [file MOL2-20-1140-s001.zip › mol270182-sup-0002-FigureS2.pdf]

**Figure S3**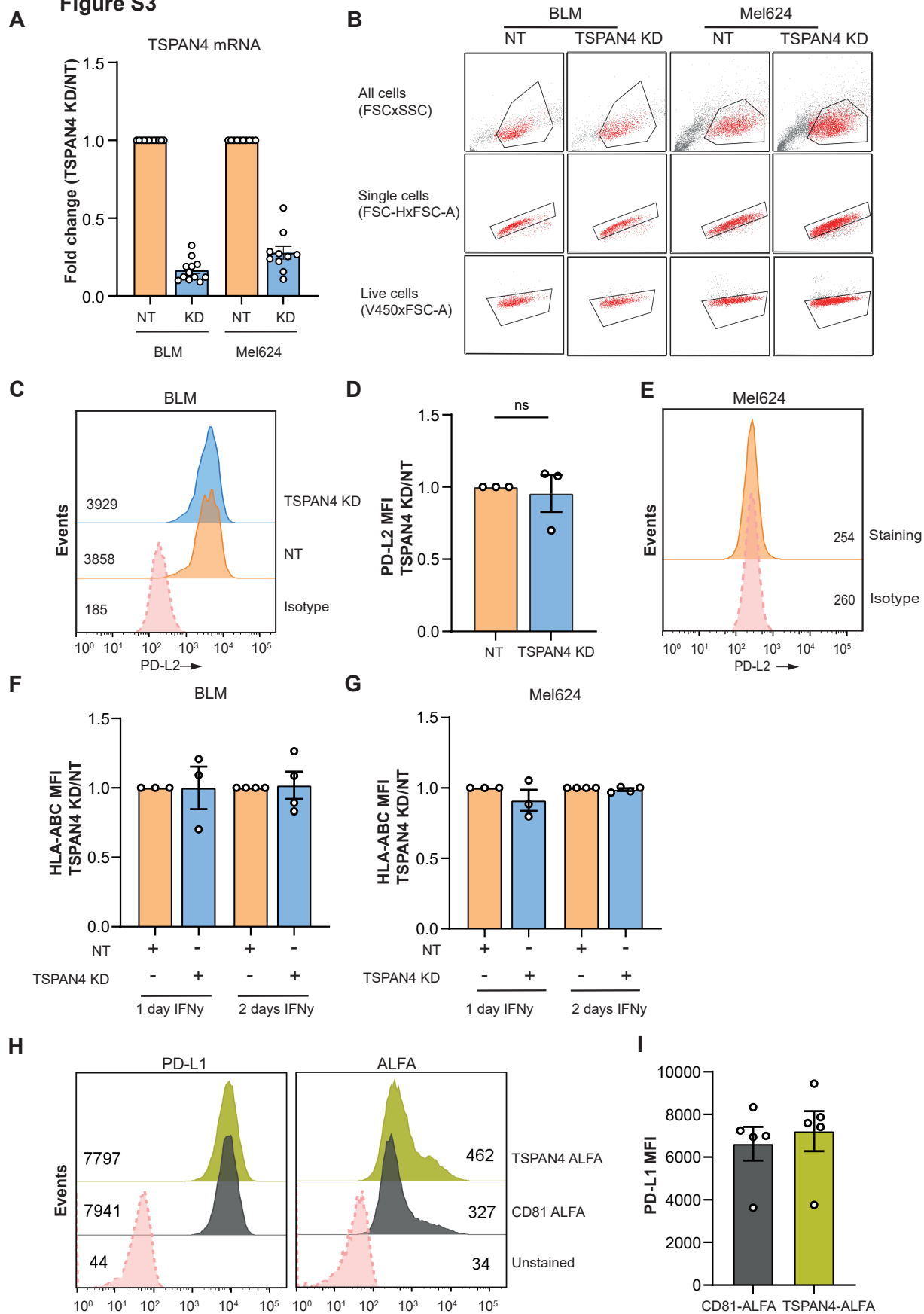

Supplement: Supplementary file 1 — Fig. S1. Identification of PD‐L1 interacting proteins in melanoma by proximity biotinylation followed by mass spectrometry. Fig. S2. Identification of PD‐L1 interacting proteins in melanoma by proximity biotinylation followed by mass spectrometry. Fig. S3. TSPAN4 negatively regulates PD‐L1 expression at the cell surface. Fig. S4. TSPAN4 mediates PD‐L1 degradation by competing with CMTM6 binding to PD‐L1. Fig. S5. TSPAN4 impacts PD‐1 binding and T cell activation. Fig. S6. Raw western blot membranes. Table S1. Mass‐spectrometry data of the PD‐L1 proximity biotinylation assay in BLM WT vs BLM PD‐L1 KO cells, with all differential hits of WT vs PD‐L1 KO cells arranged by fold difference (log2), with significance indicated. Table S2. The top 50 significantly enriched hits in WT cells from the mass‐spectrometry data of the PD‐L1 proximity biotinylation assay that were taken for GO analysis. Table S3. GO analysis of the top 50 significantly enriched hits in WT cells from the mass‐spectrometry data of the PD‐L1 proximity biotinylation assay, with false discovery rate, cellular compartment, and proteins assigned to these cellular compartments indicated. [file MOL2-20-1140-s001.zip › mol270182-sup-0003-FigureS3.pdf]

Figure S4

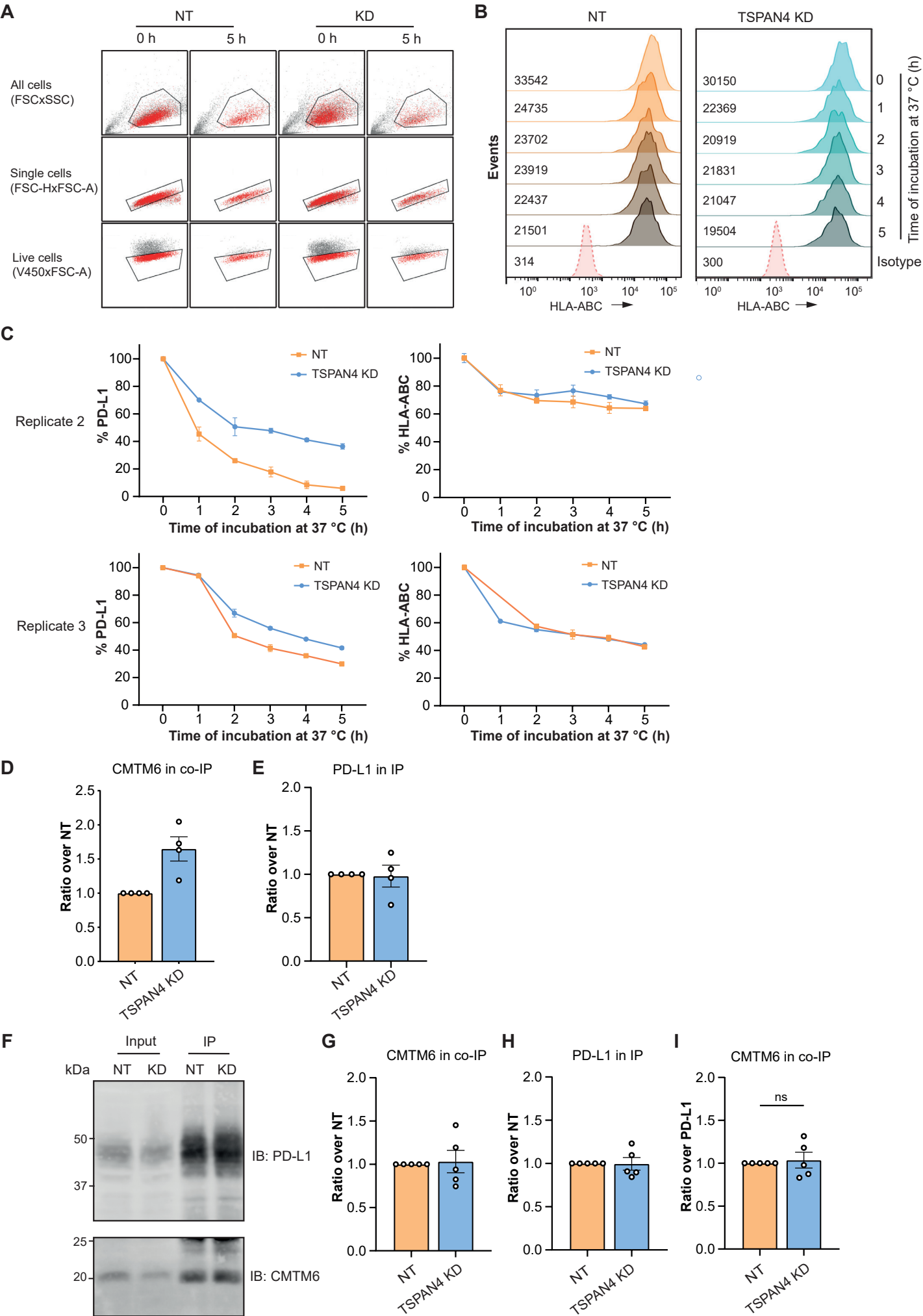

Supplement: Supplementary file 1 — Fig. S1. Identification of PD‐L1 interacting proteins in melanoma by proximity biotinylation followed by mass spectrometry. Fig. S2. Identification of PD‐L1 interacting proteins in melanoma by proximity biotinylation followed by mass spectrometry. Fig. S3. TSPAN4 negatively regulates PD‐L1 expression at the cell surface. Fig. S4. TSPAN4 mediates PD‐L1 degradation by competing with CMTM6 binding to PD‐L1. Fig. S5. TSPAN4 impacts PD‐1 binding and T cell activation. Fig. S6. Raw western blot membranes. Table S1. Mass‐spectrometry data of the PD‐L1 proximity biotinylation assay in BLM WT vs BLM PD‐L1 KO cells, with all differential hits of WT vs PD‐L1 KO cells arranged by fold difference (log2), with significance indicated. Table S2. The top 50 significantly enriched hits in WT cells from the mass‐spectrometry data of the PD‐L1 proximity biotinylation assay that were taken for GO analysis. Table S3. GO analysis of the top 50 significantly enriched hits in WT cells from the mass‐spectrometry data of the PD‐L1 proximity biotinylation assay, with false discovery rate, cellular compartment, and proteins assigned to these cellular compartments indicated. [file MOL2-20-1140-s001.zip › mol270182-sup-0004-FigureS4.pdf]

### Figure S5

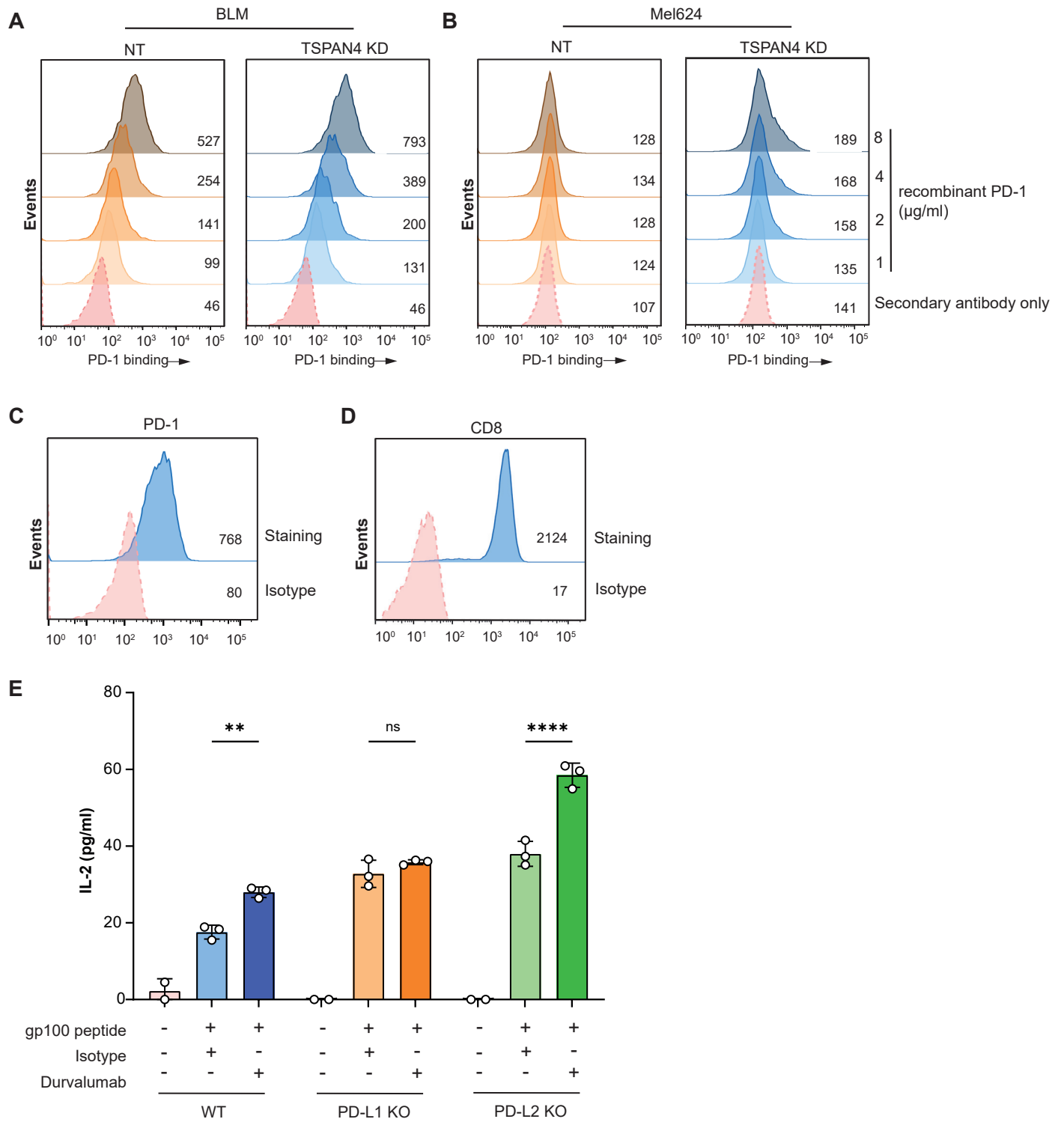

Supplement: Supplementary file 1 — Fig. S1. Identification of PD‐L1 interacting proteins in melanoma by proximity biotinylation followed by mass spectrometry. Fig. S2. Identification of PD‐L1 interacting proteins in melanoma by proximity biotinylation followed by mass spectrometry. Fig. S3. TSPAN4 negatively regulates PD‐L1 expression at the cell surface. Fig. S4. TSPAN4 mediates PD‐L1 degradation by competing with CMTM6 binding to PD‐L1. Fig. S5. TSPAN4 impacts PD‐1 binding and T cell activation. Fig. S6. Raw western blot membranes. Table S1. Mass‐spectrometry data of the PD‐L1 proximity biotinylation assay in BLM WT vs BLM PD‐L1 KO cells, with all differential hits of WT vs PD‐L1 KO cells arranged by fold difference (log2), with significance indicated. Table S2. The top 50 significantly enriched hits in WT cells from the mass‐spectrometry data of the PD‐L1 proximity biotinylation assay that were taken for GO analysis. Table S3. GO analysis of the top 50 significantly enriched hits in WT cells from the mass‐spectrometry data of the PD‐L1 proximity biotinylation assay, with false discovery rate, cellular compartment, and proteins assigned to these cellular compartments indicated. [file MOL2-20-1140-s001.zip › mol270182-sup-0005-FigureS5.pdf]
